# A general methodology to measure the light-to-heat conversion efficiency of solid materials

**DOI:** 10.1038/s41377-023-01167-6

**Published:** 2023-05-17

**Authors:** Kai Gu, Haizheng Zhong

**Affiliations:** Beijing Key Laboratory of Nanophotonics & Ultrafine Optoelectronic Systems, School of Materials Sciences & Engineering, Beijing Institute of Technology, 100081 Beijing, China

**Keywords:** Optical metrology, Nanoparticles

## Abstract

Light-to-heat conversion has been intensively investigated due to the potential applications including photothermal therapy and solar energy harvesting. As a fundamental property of materials, accurate measurement of light-to-heat conversion efficiency (LHCE) is of vital importance in developing advanced materials for photothermal applications. Herein, we report a photothermal and electrothermal equivalence (PEE) method to measure the LHCE of solid materials by simulating the laser heating process with electric heating process. The temperature evolution of samples during electric heating process was firstly measured, enabling us to derive the heat dissipation coefficient by performing a linear fitting at thermal equilibrium. The LHCE of samples can be calculated under laser heating with the consideration of heat dissipation coefficient. We further discussed the effectiveness of assumptions by combining the theoretical analysis and experimental measurements, supporting the obtained small error within 5% and excellent reproducibility. This method is versatile to measure the LHCE of inorganic nanocrystals, carbon-based materials and organic materials, indicating the applicability of a variety of materials.

## Abstract (web summary)

A general methodology was proposed to measure the light-to-heat conversion efficiency of solid materials by considering laser heating process with electric heating process.

## Graphical abstract

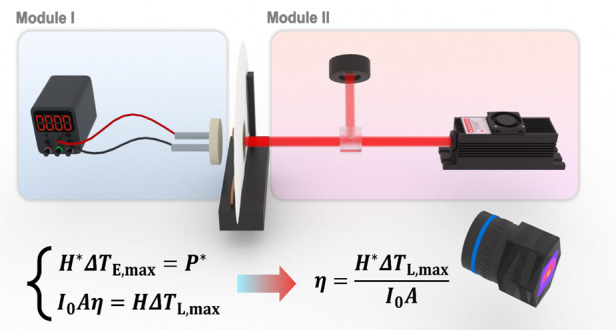

## Introduction

Light-to-heat conversion is a widespread energy conversion process in nature and human-developed systems. Photothermal materials, that include carbon-based materials^1–3^, nanocrystals^4–9^, metal oxides^10–12^ and organic molecules^13–19^, are key components to achieve functionality of photothermal therapy^20–23^, solar-driven water evaporation^24–28^, photothermal catalysis^29,30^, photothermal imaging and sensor^31–33^. Light-to-heat conversion efficiency (LHCE) is the most important figure of merit for evaluating photothermal materials^16,17^. Therefore, it has been of great interest to develop a methodology for measuring LHCE.

Based on the heat balance equation, a few methodologies have been successfully developed to measure LHCE of colloidal nanocrystal solution (solution method). For example, Roper et al. reported a cuvette model to measure LHCE of gold nanocrystal solution by monitoring temperature evolution under light irradiation with a thermocouple (Fig. S1a)^34^. To increase the heating rate of samples, Richardson et al. proposed a droplet model in which the temperature change of a gold nanocrystal droplet was directly measured to calculate the LHCE (Fig. S1b)^35^. Although, these methods have been further improved by other researchers (A review of these models can be found in Supplementary Information)^36–38^, the accuracy is still on debate due to overestimation of the mass term of measurement system as well as the imprecision fitting of heat dissipation coefficient^6,39,40^. In addition, most of the reported methodologies are limited to colloidal solutions. Therefore, it is an urgent need to develop an accurate measurement method of LHCE for solid materials.

In this work, a general methodology to measure the LHCE of solid materials was reported. We propose a photothermal and electrothermal equivalence (PEE) method that simulates the laser heating process with electric heating process. In electrothermal measurement, the heat dissipation coefficient of the sample can be derived under a known electric power by performing a linear fitting at thermal equilibrium. In photothermal measurement, the maximum temperature change of the sample is monitored under laser heating to calculate the LHCE. The versatility of the PEE method was further investigated for various solid materials. In addition, we discussed the error and reliability of the PEE method, as well as the deviations from the consideration of our assumptions in the measurement and data analysis.

## Results

### Modeling

**Fig. 1:**
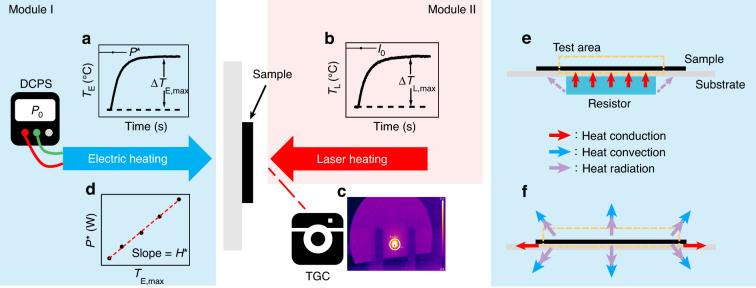
Schematic diagram of the PEE method Schematic illustrations of the temperature rise curves of a sample obtained from the TGC during electric heating (**a**) and laser heating (**b**). **c** A typical temperature image of a sample captured by the TGC. **d** Schematic illustration of the maximum temperature evolution with thermal power received by the sample. **e** Schematic diagram of the heat conduction of a sample during electric heating, where heat radiation from the side of the resistor is ignored. **f** Schematic diagram of the heat conduction, heat convection, and heat radiation of a sample in both electrothermal and photothermal measurements

Figure 1 schematically describes the experimental measurements and data analysis of PEE method. Electrothermal measurement was accomplished by determining the temperature increase of the sample on a resistor using a thermographic camera (TGC). Similarly, photothermal measurement was accomplished by determining temperature increase of the sample under laser heating. By plotting the curve of temperature evolution versus time (Fig. 1a, b), the maximum temperature change can be obtained by monitoring the average temperature using TGC (Fig. 1c). Heat dissipation coefficient of the sample can be derived by linearly fitting the plots of the maximum temperature change and the input power of electric heating (Fig. 1d). With the considerations of light absorbance and heat dissipation coefficient, the LHCE of the sample can be calculated using heat balance equation with laser heating.

Fig. S2 shows photographs of the experimental setups, where it is placed in a sealed working chamber to avoid the influence of accidental airflow. The experimental setups are divided into an electrothermal module (Module I) and a photothermal module (Module II). The electrothermal module contains a resistor, a DC power supply (DCPS), and a TGC, while the photothermal module contains a laser light source, an optical power meter, and the TGC. Samples were prepared by drop coating the sol or nanocrystal solution onto the center of filter paper to form compact structures (Fig. S3). Details of the experimental setup and sample preparation can be found in Methods.

Prior to describing the model, it is important to understand the underlying assumptions of PEE method. The main assumptions are summarized in the following. (i) As shown in Fig. 1e, the heat conduction from the resistor to the sample is the denominate pathway of heat transfer. Because the sample is closely attached to a very thin resistor, the influence of heat radiation from the side of the resistor is ignored during electric heating measurement. (ii) The heat dissipation power of the sample is simplified to be linearly increase with temperature difference in three heat transfer ways, as shown in Fig. 1f. (iii) An average temperature in the test area is used to calculate LHCE due to the inhomogeneous temperature distribution of the sample.

All the instruments should be calibrated before measurements. A typical measurement involves two steps. In module I, a sample was heated by a resistor with different input powers (*P*_0_) controlled by the DCPS. The average temperature *T*_E_ (the subscript letter E indicates electric heating) of the test area was monitored using a TGC. Figure 1a plots the recorded *T*_E_ versus time. A typical temperature photograph captured by the TGC is shown in Fig. 1c, and the test area is highlighted with a white circle. The size of the test area was calculated by measuring line temperature distribution (Fig. S4).

The heat dissipation coefficient of the sample is derived in thermal equilibrium from Eqs. 1–3.

1 $$\sum _{j}{Q}_{j}=m{c}_{p}\frac{{dT}}{{dt}}$$

2 $${P}^{* }-{H}^{* }\varDelta {T}_{{\rm{E}},{{\max }}}=0$$

3 $${P}^{* }={P}_{{\rm{un}}}=\frac{{S}_{{\rm{un}}}}{S}{P}_{0}=\frac{r}{2r+2h}{P}_{0}$$

where the energy term *Q*_*j*_ includes the input and output energy to the sample, *m* and *c*_*p*_ are the mass and heat capacity of the sample, respectively, *T* is the temperature of the sample, *t* is the measurement time, P* is the thermal power received by the sample, H* is the heat dissipation coefficient of the sample during electric heating, $\triangle {T}_{{\rm{E}},{{\max }}}={T}_{{\rm{E}},{{\max }}}-{T}_{0}$, ${T}_{{\rm{E}},{{\max }}}$ is the maximum average temperature change within the test area during electric heating, *T*_0_ is the initial temperature of the system, *P*_un_ is the output power of the resistor in the sample plane, *S*_un_ represents the contact area between resistor and sample, *S* is the surface area of the resistor, *P*_0_ is the input power of the resistor, *r* is the radius of the contact area, and *h* is the thickness of the resistor.

Equation 1 describes the general heat balance equation of a sample^41^. Equation 2 describes the heat balance equation of the sample under thermal equilibrium state. Equation 3 describes the relationship between thermal power received by the sample (P*), size of the resistor (*r* and *h*) and input power of the resistor (*P*_0_). In Eq. 2, heat conduction (Eq. 4), heat convection (Eq. 5) and heat radiation terms (Eq. 6) are considered. According to assumption (ii), heat dissipation power of the sample is simplified as linearly increase with temperature difference. In our calculation, the heat dissipation coefficient (H*) includes coefficients of heat conduction, heat convection and heat radiation (Eq. 7). The heat dissipation term (${H}^{* }\varDelta {T}_{{\rm{E}},{{\max }}}$) of Eq. 2 is proportional to the variation of temperature ($\varDelta {T}_{{\rm{E}},{{\max }}}$).

4 $${Q}_{{\rm{cond}}}={H}_{{\rm{cond}}}\varDelta {T}_{{\rm{E}},{{\max }}}$$

5 $${Q}_{{\rm{conv}}}={H}_{{\rm{conv}}}\varDelta {T}_{{\rm{E}},{{\max }}}$$

6 $${Q}_{{\rm{rad}}}={H}_{{\rm{rad}}}\varDelta {T}_{{\rm{E}},{{\max }}}$$

7 $${H}^{* }={H}_{{{cond}}}+{H}_{{{conv}}}+{H}_{{{rad}}}$$

where *Q*_cond_, *Q*_conv_, and *Q*_rad_ are the energies of heat conduction, heat convection, and heat radiation of the sample, respectively; *H*_cond_, *H*_conv_, and *H*_rad_ are the coefficients of heat conduction, heat convection, and heat radiation of the sample, respectively.

Fig. S5 shows photographs of the resistor, where the resistor is a thin sheet with a bottom diameter of 5 mm and a thickness of 0.8 mm. In fact, the sample received the heat radiation power from the side of the resistor is much smaller than the heat conduction power from the bottom of the resistor. Therefore, the heat radiation power on the side of the resistor is neglected (assumption i). According to Eq. 2, the variation of input power results in a linear fitting to obtain the heat dissipation coefficient (H*), as shown in Fig. 1d.

In module II, the sample was heated by laser irradiation. As shown in Fig. 1b, the average temperature *T*_L_ (the subscript letter L indicates laser heating) of the test area was monitored using the same TGC, and the incident laser power was obtained by an optical power meter. The test area under laser heating was fixed with the same size and position of electric heating, as shown in Fig. S6. The LHCE of the sample can be derived from Eqs. 8–11.

8 $${I}_{0}A\eta -H\varDelta {T}_{{\rm{L}},{{\max }}}=0$$

9 $$A=1-{T}^{* }-R$$

10 $$H={H}^{* }$$

11 $$\eta =\frac{{H}^{* }\varDelta {T}_{{\rm{L}},{{\max }}}}{{I}_{0}A}$$

where *I*_0_ is the incident laser power, *A* is the absorbance of the sample, *R* and T* are the reflectance and transmittance of the sample measured by a spectrophotometer, *η* is the LHCE of the sample, *T*_L_ is the average temperature of the test area during laser heating, *H* is the heat dissipation coefficient of the test area during laser heating, and $\triangle {T}_{{\rm{L}},{{\max }}}={T}_{{\rm{L}},{{\max }}}-{T}_{0}$.

Equation 8 describes the heat balance equation of the sample under laser heating, which corresponds to Eq. 2. The absorbance of the sample is calculated by Eq. 9, where reflectance and transmittance are measured using a spectrophotometer. Because the sample and substrate do not change during laser and electric heating, the temperature-independent heat dissipation coefficients are equivalent for laser (*H*) and electric heating (H*), as described in Eq. 10. Since the test system undergoes a prolonged thermal equilibrium at the beginning, the initial temperature of the filter paper and the air are both the same as *T*_0_. Consequently, the LHCE of the sample can be calculated using Eq. 11. The details of the experimental instructions can be found in Supplementary Information.

### Measurements

**Fig. 2:**
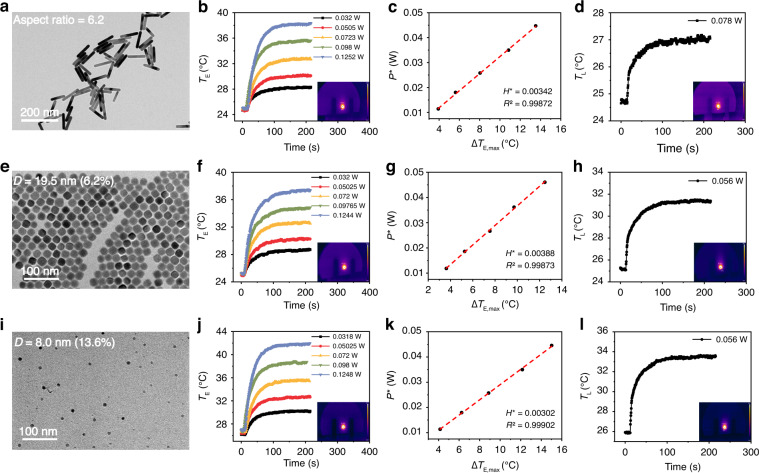
LHCE calculations of Au nanorods, PbSe nanocrystals, and Cu_2_Se nanocrystals. LHCE calculations of Au nanorods, PbSe nanocrystals, and Cu_2_Se nanocrystals. TEM images of Au nanorods (**a**), PbSe nanocrystals (**e**), and Cu_2_Se nanocrystals (**i**). Temperature evolution curves of Au nanorods, PbSe nanocrystals, and Cu_2_Se nanocrystals at different electric powers (**b**, **f**, **j**) and 980 nm laser power (**d**, **h**, **l**). The insets are the temperature photos captured by the TGC. Linear fitting curves of Au nanorods (**c**), PbSe nanocrystals (**g**), and Cu_2_Se nanocrystals (**k**) at different electric powers to calculate H*

By applying the PEE method, we measured the LHCE of Au nanorods, PbSe, and Cu_2_Se nanocrystals. Transmission electron microscopy (TEM) of Au nanorods is shown in Fig. 2a, where the statistical aspect ratio of Au nanorods is 6.2 (Fig. S7a). Figure 2b plots the temperature increase of Au nanorods under electric heating with different input powers of 0.032 W, 0.0505 W, 0.063 W, 0.0723 W, 0.098 W, and 0.1252 W. The inset is a typical temperature photograph captured by the TGC. The heat-up time is defined as the temperature change from 0 to 90% and the heat-up time of Au nanorods is about 76 seconds. According to Eq. 2, the H* of Au nanorods can be derived and the result is shown in Fig. 2c. The correlation coefficient (R^2^) of the fitting is 0.998, indicating the effectiveness of assumption (ii). Figure 2d shows the temperature increase of Au nanorods under 980 nm laser heating with incident laser power of 0.078 W. The $\triangle {T}_{{\rm{L}},{{\max }}}$ of Au nanorods is 2.4 °C and the heat-up time of laser heating is about 67 seconds, which is much shorter than that in solution method (~400 seconds)^34^. The transmission and reflectance curves of the Au nanorods are shown in Fig. S8a. According to Eq. 11, the calculated LHCE of Au nanorods is 67.4%.

Similarly, we also measured the LHCE of PbSe and Cu_2_Se nanocrystals. As shown in Fig. 2e, i, the samples have an average diameters of 19.5 nm and 8.0 nm for PbSe and Cu_2_Se nanocrystals, respectively. X-ray diffraction (XRD) exhibits that the synthesized PbSe and Cu_2_Se nanocrystals are cubic phase and α-phase, respectively (Fig. S9). Figure 2f, j show the temperature increase of samples under electric heating with different input powers, and Fig. 2g, k show the derived H* of the samples. Figure 2h, i show the temperature increase of samples under 980 nm laser heating. The calculated LHCE of the PbSe and Cu_2_Se nanocrystals are 91.9% and 71.0%, respectively.

**Fig. 3:**
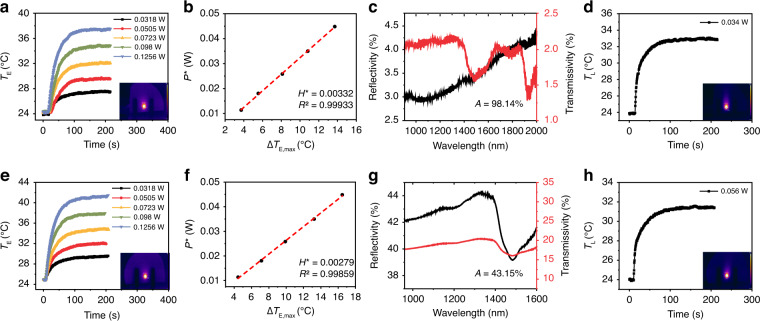
LHCE calculations of MWCN and PANI. Temperature evolution curves of MWCN (**a**, **d**) and PANI (**e**, **h**) at different electric powers and 980 nm laser power. The insets are the temperature photos captured by the TGC. Linear fitting curves of MWCN (**b**) and PANI (**f**) at different electric powers to calculate H*. Reflection and transmission spectra of MWCN (**c**) and PANI (**g**) on the filter paper

To demonstrate the applicability of the PEE method, the LHCE of carbon-based materials and polymers were also measured, including multi-walled carbon nanotube dispersions (MWCN) (Fig. 3a–d), graphene oxide sols (GO) (Fig. S10a–d), graphene dispersions (Fig. S10e–h) and polyaniline (PANI) (Fig. 3e–h). The differential scanning calorimetry (DSC) curves, as shown in Fig. S11, indicate that the samples do not undergo any phase change during temperature increasing.

## Discussion

### Error and reliability

**Fig. 4:**
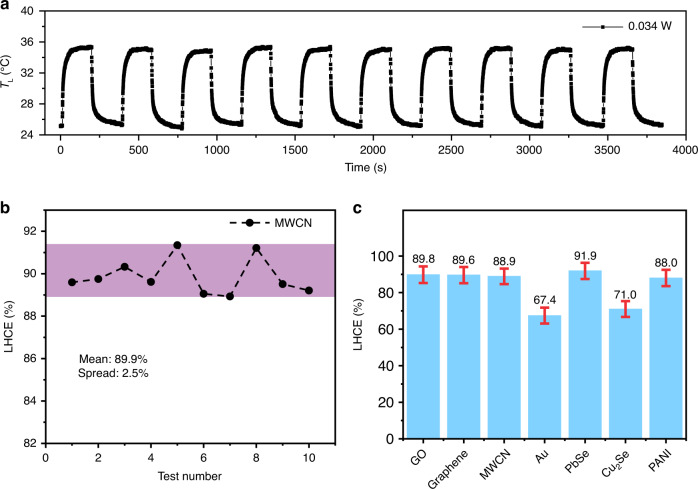
Summary of LHCE for all samples. Temperature evolution of MWCN under 980 nm continuous laser heating (**a**) and the corresponding calculated LHCE (**b**). **c** The calculated LHCE of all samples, where error bars were derived from the eq. S15

To analyze the error and statistical spread in the PEE method, the temperature evolutions of MWCN (Fig. 4a), PbSe, and Au nanorods (Fig. S12) during ten tests were recorded under continuous laser heating. The maximum temperature differences (${\Delta T}_{{\rm{L}},{{\max }}}$) of the sample were almost kept the same with the heat-up and cool-down periods fixed to 180 seconds. An average LHCE was calculated to be 89.9% with a spread of 2.5% for MWCN (Fig. 4b). We further evaluated the deviation of the PEE method by taking total differential of eq. S15, where the variation of absorbance of samples with temperature was considered (Fig. S13). The calculated LHCE of all samples are shown in Fig. 4c. The deviations of all samples are within 5%, which is a great improvement in comparison with previous solution method (~39%)^40^. Furthermore, we compared the LHCE measurements of Au nanorods in solid and aqueous solution using the PEE method and previous solution method (Fig. S14). The average LHCE of Au nanorods is 67.7 ± 11.5% for solution method and 67.4 ± 3.29% for the PEE method. A spread of 29.7% is observed for the measured LHCE using solution method, while the PEE method only shows a spread of 3.3%. These results confirmed the reliability and advantages of PEE method in determining the LHCE of photothermal materials.

### Analysis of model assumptions

**Fig. 5:**
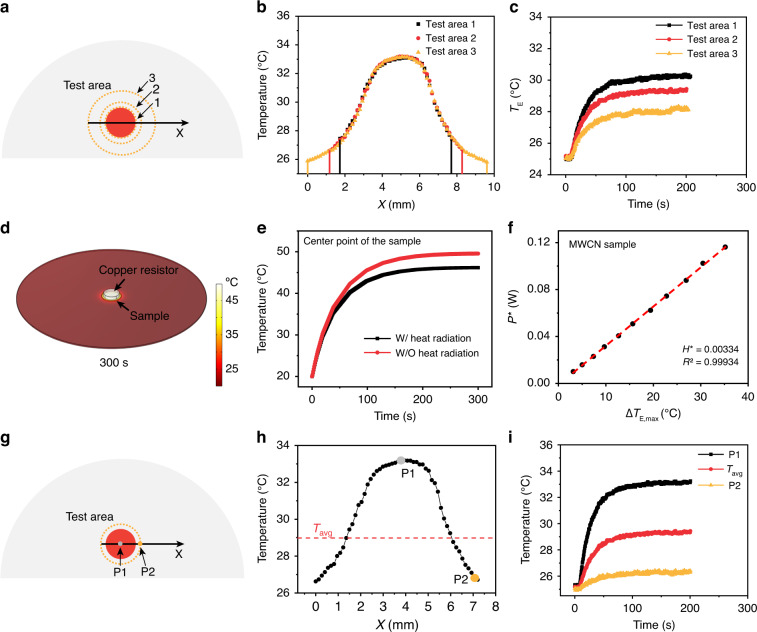
Analysis of model assumptions. **a** Schematic diagram of the heated MWCN sample monitored using different sizes of test areas. **b** The line temperature distribution of different sizes of test areas along the X-direction. **c** The average temperature increase curves of the different sizes of test areas. **d** Simulation diagram of the temperature distribution of the system under electric heating. **e** Temperature evolution curves at the center point of the sample with and without considering heat radiation. **f** Measured P* and $\triangle {T}_{{\rm{E}},{\rm{\max }}}$ relationship curve of the MWCN sample. **g** Schematic diagram of the heated MWCN sample monitored using single point temperature and average temperature. **h** The line temperature distribution along the X-d**i**rection. **i** The temperature increase curves of P1, P2, and *T*_avg_

To clarify the influence of heat radiation from the side of the resistor in assumption (i), we varied the size of the test area to alternate heat radiation to the sample. As shown in Fig. 5a, b, three different sizes of test areas were selected to calculate the LHCE of MWCN sample, where the diameter of the test area 3 and test area 2 are 1.6 times and 1.2 times than that of the test area 1. Figure 5c shows the variation of average temperature with time in different test areas. The calculated LHCE is 90.8% for the test area 1, 90.3% for the test area 2 and 83.1% for the test area 3 (calculation details are shown in Table S1). These results indicate that the selection of a large test area can cause a deviation from the assumption (i) in the PEE method, which can be explained to the underestimation of P* due to the inappropriate neglecting of heat radiation from the side of the resistor to the sample.

To clarify the validity of assumption (ii), we here discuss the influence of heat radiation on the calculation of LHCE. We firstly simulated the influence of heat radiation on the temperature increasing during electric heating process. The modeling details are provided in Method. Figure 5d shows the simulated temperature distribution of the system at 300 seconds under electric heating. Figure 5e shows the temperature evolution with time prolonging at center point of the sample with and without consideration of the heat radiation effects. Therefore, the proportion of heat radiation to heat dissipation can be calculated as 10.3% based on the temperature difference, which is similar to the value that determined from experimental data (11%, see Supplementary Information). According to the Stefan-Boltzmann law^42^, Eq. 7 can be rewritten as Eq. 12. The relative rate of change for H* is calculated to be 0.05% using Eq. 13, in the temperature change range of 0~50 °C, as shown in Fig. S15. Therefore, the influence of H* with temperature is negligible, suggesting the validity of assumption (ii). This conclusion is also supported by the experimental results. As shown in Fig. 5f, the linearly relationship between P* and $\triangle {T}_{{\rm{E}},{\rm{\max }}}$ of MWCN can be well kept when the temperature change increasing up 40 °C. In addition, it is suggested that the diameter of the resistor should be less than 20 mm to minimize the influence on H* (Fig. S16).

12 $${H}^{* }\left(T\right)={H}_{{\rm{cond}}}+{H}_{{\rm{conv}}}+s\varepsilon \sigma \left({T}^{2}+{T}_{0}^{2}\right)\left(T+{T}_{0}\right)$$

13 $$\frac{d{H}^{* }}{{dT}}/{H}^{* }=\frac{s\varepsilon \sigma \left(3{T}^{2}+2{T}_{0}T+{T}_{0}^{2}\right)}{{H}^{* }}$$

where *s* is the area of the test area including both the front and back sides, *ε* is the emissivity of the sample and *σ* is the Stefan-Boltzmann constant.

For assumption (iii), we further analyzed the difference of LHCE measurements using single point temperature and average temperature. Figure 5g, h show the line temperature distribution along the X-direction of the MWCN sample in electric heating process. We selected two typical points (P1 and P2) in the test area and measured the temperature increase curves of these two points, as shown in Fig. 5c. In addition, *T*_avg_ was denoted as the average temperature of the test area. The inhomogeneous temperature distributions of the sample were also observed for both electric and laser heating (Fig. S17). Table S2 summarized the calculated LHCE using the temperature of P1 (160.0%), the temperature of P2 (143.2%), and the average temperature (88.9%), respectively. It is noticed that the calculated LHCE using the single point temperature of P1 or P2 are unreasonable. Moreover, due to the thickness of the substrate (~0.34 mm), the temperature difference in the vertical direction of the substrate under laser and electric heating is negligible (Fig. S18). Therefore, an accurate LHCE is obtained by considering the average temperature of the sample.

In conclusion, we demonstrated a PEE method to measure the LHCE of solid-state materials by simulating laser heating with electric heating. The heat dissipation coefficient of the sample under electric heating is equal to that under light heating, which can be obtained by linearly fitting the maximum temperature change and input power under electric heating. Therefore, the LHCE of the sample under laser heating can be directly derived using heat balance equation. To demonstrate the applicability of the PEE method, we measured the LHCE of various organic and inorganic photothermal materials. The measured LHCE is 89.8% ± 4.55% for GO, 89.6% ± 4.44% for graphene, 88.9% ± 4.25% for MWCN, 67.4% ± 4.35% for Au nanorods, 91.9% ± 4.43% for PbSe nanocrystals, 71.0% ± 4.34% for Cu_2_Se nanocrystals and 88.0% ± 4.46% for PANI. Furthermore, we discussed the error and reliability of the PEE method and deviations from assumptions to demonstrate the advantages of the PEE method. In all, this work provides a convenient methodology to measure the LHCE, which have potential to promote the fundamental research of advanced photothermal materials.

## Materials and methods

### Chemicals

All the chemicals were used without further purification. Lead (II) oxide (PbO, 99.9%, Aladdin), selenium powder (Se, 99.99%, Aladdin), copper sulfate pentahydrate (CuSO_4_·5H_2_O, 99.9%, Aladdin), selenium dioxide (SeO_2_, 99.999%, Macklin), ascorbic acid (Vc, >99%, Sigma-Aldrich), oleic acid (OA, 90%, Aladdin), trioctylphosphine (TOP, 90%, Aladdin), 1-octadecene (ODE, 90%, Aladdin), oleylamine (OLA, 80~90%, Aladdin), triphenylphosphine (TPP, 99%, Macklin), hexane (99%, Sigma-Aldrich), methanol (anhydrous, 99.5%, Sigma-Aldrich), N,N-Dimethylformamide (DMF, anhydrous, 99.8%, Sigma-Aldrich), graphene oxide gel (GO, 1~3 wt%, Aladdin), graphene solution (0.4~0.6 wt%, Aladdin), Multi-Walled carbon nanotubes dispersion (MWCN, 2~3 wt%, Macklin), gold nanorods (XFNANO materials Tech Co.), and polyaniline (PANI, 98%, Macklin).

### Synthesis of PbSe nanocrystals

In a typical synthesis, TOPSe (selenium powder dissolved in TOP) was used as the molecular precursors by heating TOP and Se powder at 40 °C for 30 min. 1.116 g of PbO, 7.05 mL of OA, and 38.5 mL of ODE were degassed under vacuum at 100 °C for an hour until a light yellow clarification solution achieved. Then the solution was heated to 240 °C under flowing nitrogen, at which point 2.5 mL of a 2 M solution of TOPSe was rapidly injected to trigger the nucleation. After an hour, the reaction was quenched by a water bath. The obtained nanocrystals were purified by three rounds of precipitation−redispersion with methanol/hexane, dried completely, and dispersed in hexane.

### Synthesis of Cu_2_Se nanocrystals

A modified method was used to synthesize Cu_2_Se nanocrystals based on ref. ^43^. The Se precursor was prepared by mixing 1 mL of 0.2 M solution of SeO_2_ dissolved in deionized water and 1 mL of 0.4 M solution of Vc dissolved in DMF with continuous stirring at room temperature. The preparation of Cu precursor is similar to that of Se precursor. 0.8 mmol of TPP was added into 2 mL of 0.4 M of CuSO_4_-DMF solution. The solution was then injected to 2.5 mL of 0.4 M Vc-DMF to obtain the Cu precursor. Cu_2_Se nanocrystals were synthesized by directly mixing the Se precursor with Cu precursor for 1.5 h reaction at room temperature. After centrifugation of the above Cu_2_Se solution, the supernatant was discarded and the precipitated Cu_2_Se nanocrystals were dispersed into DMF.

### Sample preparation of PEE method

Filter paper is selected as a substrate due to its good permeability to the nanocrystal solution as well as the good transparency to 980 nm light. Fig. S19a shows the scanning electron microscopy (SEM) and a photograph of the filter paper. Fig. S19b shows that the raw filter paper does not exhibit any absorption of 980 nm light. As shown in Fig. S19c, the raw filter paper does not experience any phase change (sapphire standard sample for comparison) in the temperature range between 0~100 °C.

The medium-speed qualitative filter papers (*ф* = 110 mm, grade 102) used as supports were required to be sufficiently dried before the sample preparation. A few drops of sol or dispersion, such as GO and nanocrystals solution, were dropped on a filter paper and then heated at 60 °C to remove residual solvent. PANI powder was dissolved into DMF and then dropped onto a filter paper for sample preparation.

### Characterization

Optical transmission and reflection spectra were collected using a Lambda 1050+ UV/Vis/NIR spectrophotometer (PerkinElmer, UK) equipped with an integrating sphere. Temperature-dependent UV-Vis-NIR absorption spectra were collected by UV/Vis/NIR spectrophotometer (Shimadzu UV-3600, Japan). TEM observations were performed using a FEI Tools F200S field-emission transmission electron microscope (FEI Co., USA) operated at 200 kV. DSC measurements were carried out on Mettler DSC 3 (Mettler Toledo, Switzerland) instrument with the standard sample of sapphire for comparison. SEM measurements were performed with SU-8230 microscope (Hitachi, Ltd.) equipped with a cold field-emission gun able to accelerate the electron at 30 kV. XRD patterns were recorded by a Bruker D8 FOCUS advance X-ray diffractometer operated at 40 kV and 200 mA current under Cu Kα radiation (wavelength of 1.5418 Å).

### Simulation

We simulated the temperature change of the sample during electric heat process using COMSOL Multiphysics software. The module of the “Heat Transfer in Solids, Time Dependent” was used. The size parameters of the model are shown in Fig. S20, where the thickness of the substrate is 0.4 mm and the thickness of the resistor is 1 mm. The material properties of the substrate were referenced to that of the filter paper, including a thermal conductivity of 0.04 W m^−1^ K^−1^, density of 0.2 g cm^−3^ and heat capacity of 0.9 J g^−1^ K^−1^. The material properties of the resistor were selected as the copper in Material Library. The material properties of the test area were consistent with those of the substrate, due to the small amount of samples. The initial temperature of the system was set to 293.15 K and the heat rate of the resistor was set to 0.05 W. All surfaces, except the test area, were set up for External Natural Convection and Surface-to-Ambient Radiation conditions. For the test area, External Natural Convection was considered, however, Surface-to-Ambient Radiation was considered depending on the situation. The entire system was simulated for 300 seconds to reach thermal equilibrium.

### Experimental details

Prior to measuring LHCE, all the instruments need to be calibrated.

The laser heating module consists of the activation light source−infrared laser diode (980 nm, 2 W, Changchun New Industries Optoelectronics Tech. Co., Ltd.), two optical power meters (photodiode S120C head and PM100USB power meter, Thorlabs) which record the incident and transmission power, respectively, and the thermographic camera (FLIR A700, IR resolution 640 × 480, thermal sensitivity <30 mK). The distance between the sample and the light source is 25 cm and the diameter of light spot is ~6 mm. The distance between the TGC and the sample is 30 cm at an angle of 30 degrees from the optic axis. The electric heating module consists of the ceramic resistor (1.14 Ω) with 5 mm diameter and 0.8 mm thickness, DCPS (0~30 V), and the TGC located on the same position as the laser heating module. The resistor is pressed against the sample during the electric heating measurement. *T*_E_ and *T*_L_ are the mean temperature of the test area, which contains the main concentration of sample temperature caused by electric or laser heating, determined in the FLIR Tools software. The saturation absorptivity of sample is calculated according to Eq. 9, where T* and *R* are the transmittance and reflectivity of the sample, respectively, measured by spectrophotometer. The laser power should be sufficiently high during laser heating to ensure that the sample reaches saturation absorption, which can be determined by placing an optical power meter behind the sample to detect transmitted light.

## Supplementary information

Supplementary information
