# Supplementary material for: A general methodology to measure the light-to-heat conversion efficiency of solid materials
Source: Light Sci Appl. 2023 May 17;12:120. doi: 10.1038/s41377-023-01167-6 (PMC10188530; doi:10.1038/s41377-023-01167-6)
Supplement: Supplementary file 1 — Supplementary information [file 41377_2023_1167_MOESM1_ESM.docx]

**Supplementary Information**

**A General Methodology to Measure the Light-to-Heat Conversion Efficiency of Solid Materials**

*Kai Gu^1^, Haizheng Zhong^1,^**

^1^ Beijing Key Laboratory of Nanophotonics & Ultrafine Optoelectronic Systems, School of Materials Sciences & Engineering, Beijing Institute of Technology, 100081 Beijing, China

*e-mail: hzzhong@bit.edu.cn

**Contents**

PART 1: Previous reports of LHCE methods

PART 2: The experimental instructions of PEE method

PART 3: Evaluation of errors for PEE method

PART 4: Calculation of the heat radiation

PART 5: Figures and Tables

**PART 1: Previous reports of LHCE methods**

**1.1 Roper’s model^1^**

The energy balance in eq. 1 is simplified as

|  | $\sum_{i} m_{i}c_{p,i}\frac{dT}{dt}=Q_{I}+Q_{0}-Q_{\mathrm{ext}}$ | (S1) |
| --- | --- | --- |

where *m_i_c_p_*_,_*_i_* includes the solution and sample cell. Source term *Q*_0_ represents heat dissipated from light absorbed by the quartz sample cell itself (Fig. S1a). The laser-induced source term *Q*_I_ represents heat dissipated.

|  | $Q_{I}=I\left( 1-{10}^{-A_{\lambda}} \right)\eta$ | (S2) |
| --- | --- | --- |

where *I* is incident laser power, *η* represents the LHCE, and *A*_λ_ is absorbance of NCs given by Beer-Lambert’s Law. The external heat flow power *Q*_ext_ of the system is proportional to the temperature difference with the ambient temperature *T*_amb_, with *H* as the proportionality factor.

|  | $Q_{\mathrm{ext}}=H\left( T-T_{\mathrm{amb}} \right)$ | (S3) |
| --- | --- | --- |

In order to solve *η*, it is necessary to solve for *H*. When laser irradiation ceases, $Q_{I}+Q_{0}=0$ and the system cools, reducing eq. S1 to

|  | $\sum_{i} m_{i}c_{p,i}\frac{dT}{dt}=-H\left( T-T_{\mathrm{amb}} \right)$ | (S4) |
| --- | --- | --- |

Eq. S4 may be solved using the initial condition *T* = *T*_max_ at *t* = 0. The LHCE can be solved using equation.

|  | $\eta=\frac{H\left( T_{\max}-T_{\mathrm{amb}} \right)-Q_{0}}{I\left( 1-{10}^{-A_{\lambda}} \right)}$ | (S5) |
| --- | --- | --- |

**1.2 Richardson’s model^2^**

The calculation of LHCE in Richardson's model is similar with that of Roper's model.

|  | $m_{w}c_{p,w}\frac{dT}{dt}=Q_{I}-Q_{\mathrm{ext}}=I\left( 1-{10}^{-A_{\lambda}} \right)\eta-H\left( T-T_{\mathrm{amb}} \right)$ | (S6) |
| --- | --- | --- |

In this equation mass (*m*_w_) and heat capacity (*c_p_*_,w_) components of the system are the mass and heat capacity of the droplet, and the significance of the other variables is consistent with Roper's model (Fig. S1b). Eq. S6 can be simplified (eq. S7) by collecting terms and a variable change, *T*^*^, where *T*^*^ is the temperature difference (*T* - *T*_amb_) from the ambient temperature.

|  | $\frac{dT^{*}}{dt}=A-BT^{*}$ | (S7) |
| --- | --- | --- |

*A* and *B* are shown in eqs. S8 and S9, where *A* (°C s^-1^) is the rate of energy absorption and *B* (s^-1^) the rate constant associated with heat loss.

|  | $A=\frac{I\left( 1-{10}^{-A_{\lambda}} \right)\eta}{m_{w}c_{p,w}}$ | (S8) |
| --- | --- | --- |
|  | $B=\frac{H}{m_{w}c_{p,w}}$ | (S9) |

The heating temperature trace is given by eq. S10. The LHCE can be obtained from eq. S11, in advance of fitting eq. S10 to obtain the parameter *A*.

|  | $T\left( t \right)=T_{\mathrm{amb}}+\frac{A}{B}\left( 1-e^{-Bt} \right)$ | (S10) |
| --- | --- | --- |
|  | $\eta=\frac{A\cdot m_{w}c_{p,w}}{I\left( 1-{10}^{-A_{\lambda}} \right)}$ | (S11) |

**1.3 Wang’s model^3^**

Wang et al. argued that the mass and heat capacity terms in eq. S11 are not the product of the mass and heat capacity of the parts of the system. In the calibration experiment, the laser was replaced with a resistance, which was connected to a battery and immersed in the solution (Fig. S1c). Assuming that resistance completely converts electrical energy into heat, then eq. S8 can be rewritten when the solution is heated with the resistance:

|  | $A=\frac{P}{mc_{p}}$ | (S12) |
| --- | --- | --- |

where the power *P* is taken to be the heat power of the resistance, which is proportional to *U*^2^ and inversely proportional to *R*. In this equation, the proportionality factor *mc_p_* called the "effective mass" can be obtained from a linear fit by variation of *P*. Therefore, the LHCE can be calculated by rewriting eq. S12 to obtain.

|  | $\eta=\frac{A\cdot mc_{p}}{I\left( 1-{10}^{-A_{\lambda}} \right)}$ | (S13) |
| --- | --- | --- |

**1.4 Pasciak’s model^4^**

Pasciak et al. combined the advantages of Richardson’s and Wang's models and measured the average temperature of the droplet with a TGC while calibrating the mass term (Fig. S1d). As a result, the procedure for calculating the LHCE is identical to Wang's model, with eq. S13.

**PART 2: The experimental instructions of PEE method**

Fig. S2 shows photographs of the experimental setup, which is divided into electric (Module I) and heating module (Module II). Module I contains a resistor (A thin sheet with a bottom diameter of 5 mm and a thickness of 0.8 mm, 1.14 Ω), a DCPS (0~30 V), and a TGC (FTIR A700), while Module II contains a continuous laser (980 nm, 0~2 W), an optical power meter (PM100USB), and the same TGC. Samples were prepared by drop coating nanocrystals on filter paper with an area larger than the area of the spot of the laser source and fixed between the two modules. As shown in Fig. S3, nanocrystal samples need to be closely aligned to avoid isolated hot spots to form temperature confinement phenomena^5^. The center of the light source, the resistor and the sample are fixed on the same horizontal line. The diameter of the light spot is about 6 mm which is slightly larger than that of the resistor (5 mm) to obtain the same size of heating area (Fig. S6). According to eq. 13, Fig. S16 shows the influence of size of resistor on the validity of assumption (ii). As shown in Fig. S16c, an excellent linear relationship between the maximum temperature difference and the input power is obtained with a small resistor. Therefore, a small resistor with diameter less 20 mm is preferred for the LHCE measurements. The other experimental setups were placed in a sealed work box, except for the DCPS, laser power supply and the computer controlling the TGC. Prior to the measurement, all the instruments should be calibrated. Besides, the ambient temperature outside the work box is kept stable. The methodology is only applicable to the sample without any phase change during measurement.

The experimental steps are as follows.

1) The resistor is attached onto the filter paper with sample.

2) A fixed test area is defined on the computer to monitor the temperature change of the sample.

3) Set the power of the resistor to *P*_0_ by DCPS.

4) Start electric heating and record the average temperature in the test area.

5) When the sample reaches thermal equilibrium, stop heating and save the temperature change curve. Note: The maximum temperature ($T_{E,max}$) of the sample below 50 °C is suggested due to the demand of assumption (ii). The maximum temperature change ($\Delta T_{E,max}$) of the sample is suggested to control in the range between 3~10 °C.

6) After the temperature of the sample is stabilized with the ambient temperature, change the value of *P*_0_ and repeat steps 3-6.

7) Calculate *P** according to eq. 3 and extract the maximum average temperature change ($\Delta T_{E,max}$) from the temperature change curve.

8) According to eq. 2, a linear fit is performed using *P** and $\Delta T_{E,max}$ to obtain *H**, as shown in Fig. 1d.

9) Remove the resistor.

10) The fixed test area is used to monitor the temperature change of the sample.

11) Set the power of the laser to *I*_0_.

12) Adjust *I*_0_ to allow the laser to transmit the sample.

13) Start laser heating and record the average temperature in the test area.

14) When the sample reaches thermal equilibrium, stop heating and save the temperature change curve.

15) Extract the maximum average temperature change ($\Delta T_{L,max}$) from the temperature change curve. Note: The maximum temperature ($T_{L,max}$) of the sample below 50 °C is suggested. The maximum temperature change ($\Delta T_{L,max}$) of the sample is suggested to control in the range between 3~10 °C.

16) Measure the transmittance and reflectance of the sample using a spectrophotometer, and then calculate the absorbance of the sample according to eq. 9.

17) Finally, calculate the LHCE of the sample according to eq. 11.

**PART 3:** **Evaluation of errors for PEE method**

|  | $s_{\eta}^{2}=\left( \frac{\partial\eta}{\partial H^{*}} \right)^{2}s_{H^{*}}^{2}+2\left( \frac{\partial\eta}{\partial T_{L}} \right)^{2}s_{T_{L}}^{2}+\left( \frac{\partial\eta}{\partial A} \right)^{2}s_{A}^{2}+\left( \frac{\partial\eta}{\partial I_{0}} \right)^{2}s_{I_{0}}^{2}$ | (S14) |
| --- | --- | --- |
|  | $\frac{s_{\eta}}{\eta}=\sqrt{\left( \frac{s_{H^{*}}}{H^{*}} \right)^{2}+2\left( \frac{s_{T_{L}}}{T_{L}} \right)^{2}+\left( \frac{s_{I_{0}}}{I_{0}} \right)^{2}+\left( \frac{s_{T}}{T^{*}} \right)^{2}+\left( \frac{s_{R}}{R} \right)^{2}}$ | (S15) |

where $s_{\eta}$, $s_{H^{*}}$, $s_{T_{L}}$, $s_{I_{0}}$, $s_{A}$, $s_{T^{*}}$, $s_{R}$ are the standard deviations of the corresponding subscript physical quantity, $\frac{s_{H^{*}}}{H^{*}}$ is the relative error of the fitting, $\frac{s_{T_{L}}}{T_{L}}=2\%$, $\frac{s_{I_{0}}}{I_{0}}=0.5\%$ are the relative errors of the instruments, $\frac{s_{T}}{T^{*}}=\frac{s_{R}}{R}=2\%+0.02\%=2.02\%$ includes the error of absorbance influenced by temperature change and the relative error of the instrument.

The absorbance values of gold nanorods and carbon materials vary about ~4% from room temperature to 100 °C^6-9^. Fig. S13 shows the temperature-dependent absorbance spectra of PbSe and PANI powders. The absorbances of PbSe and PANI powders varied by ~3.9% and 3.4% at 980 nm from room temperature to 75 °C, respectively. When considering the distribution of absorbance (due to temperature distribution), the influence of absorbance with temperature increase is about ~2%, which is within the errors of the PEE measurements.

**PART 4: Calculation of the heat radiation**

The proportion of the heat radiation term ($Q_{rad}$) to the heat dissipation term ($Q_{cond}+Q_{conv}+Q_{rad}$) can be calculated by eq. S16. The calculated result is 11% similar to the simulation result (10.3%).

|  | $\frac{Q_{\mathrm{rad}}}{Q_{\mathrm{cond}}+Q_{\mathrm{conv}}+Q_{\mathrm{rad}}}=\frac{s\varepsilon\sigma\left( T^{4}-T_{0}^{4} \right)}{H^{*}\left( T-T_{0} \right)}=\frac{s\varepsilon\sigma\left( T^{2}+T_{0}^{2} \right)\left( T+T_{0} \right)}{H^{*}}$ | (S16) |
| --- | --- | --- |

where *s* is the area of the test area including both front and back sides ($18\pi\times{10}^{-6} m^{2}$), *ε* is the emissivity of the sample (take 1 for the convenience of calculation), *σ* is the Stefan-Boltzmann constant ($5.67\times{10}^{-8} W m^{-2} K^{-4}$), $T=318.15 K$, $T_{0}=293.15 K$ and $H^{*}=3.32\times{10}^{-3} W K^{-1}$ (using the *H** of MWCN).

**PART 5: Figures and Tables**

**Table S1**. Details of LCHE calculation using different sizes of test areas.

| Test area | *T*_E_ (˚C) (0.032 W) | *H** (W ˚C^-1^) | *T*_L_ (˚C) (0.034 W) | LHCE (a.u.) |
| --- | --- | --- | --- | --- |
| 1 | 5.27 | 0.00238 | 12.74 | 90.8% |
| 2 | 4.23 | 0.00298 | 10.11 | 90.3% |
| 3 | 2.89 | 0.00416 | 6.66 | 83.1% |

**Table S2**. Details of the LCHE calculation from P1, P2 and *T*_avg_.

|  | Temperature under electric power (0.032 W) | *H** (W ˚C^-1^) | Temperature under laser power (0.034 W) | LHCE (a.u.) |
| --- | --- | --- | --- | --- |
| $P1$ | 7.81 | 0.00151 | 35.35 | 160.0% |
| *T*_avg_ | 4.24 | 0.00298 | 9.96 | 88.9% |
| $P2$ | 1.22 | 0.01727 | 1.43 | 143.2% |


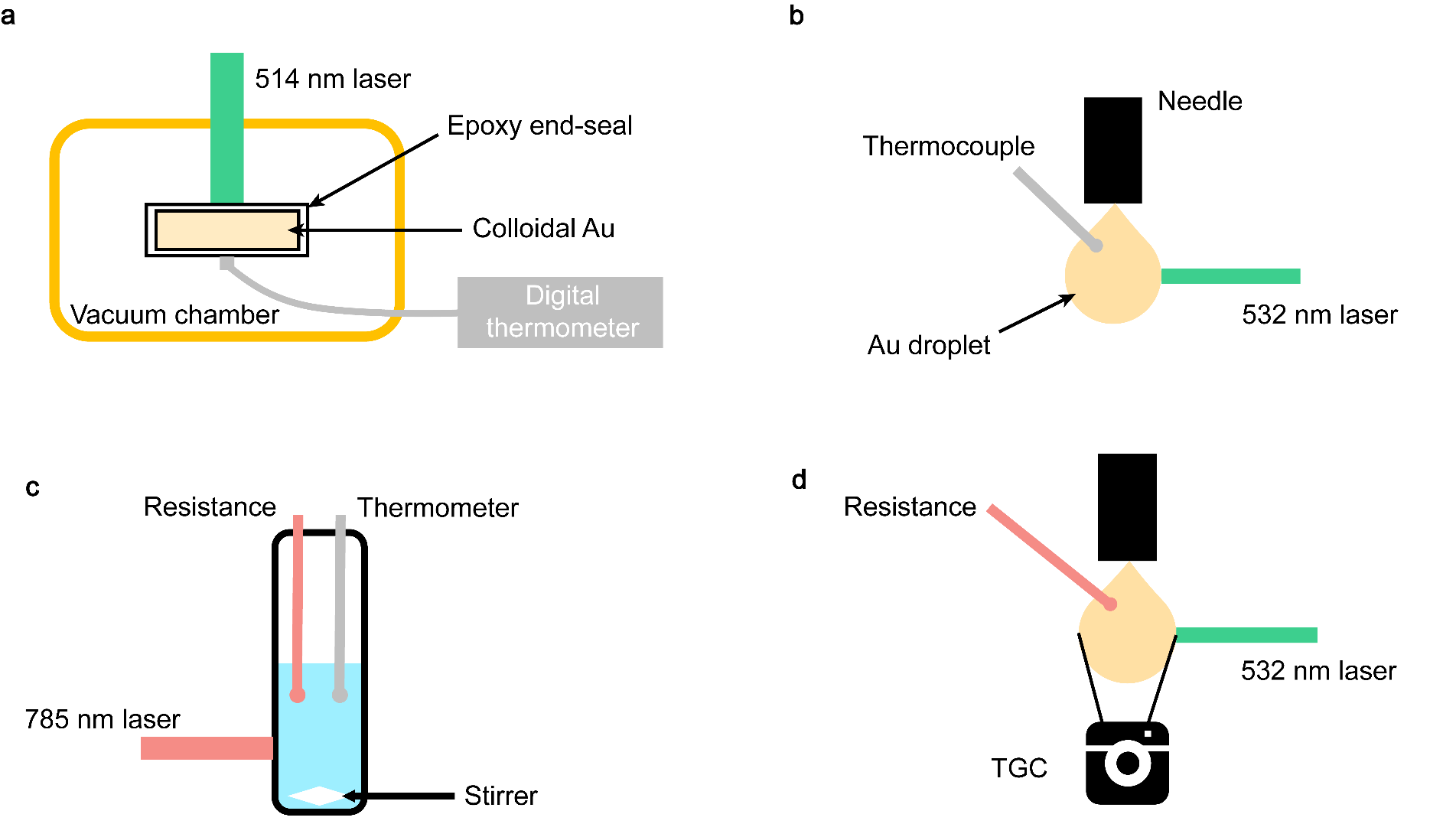


**Fig. S1.** Diagrams of the experimental setup for the LHCE measurement proposed by Roper (**a**), Richardson (**b**), Wang (**c**), and Pasciak (**d**).


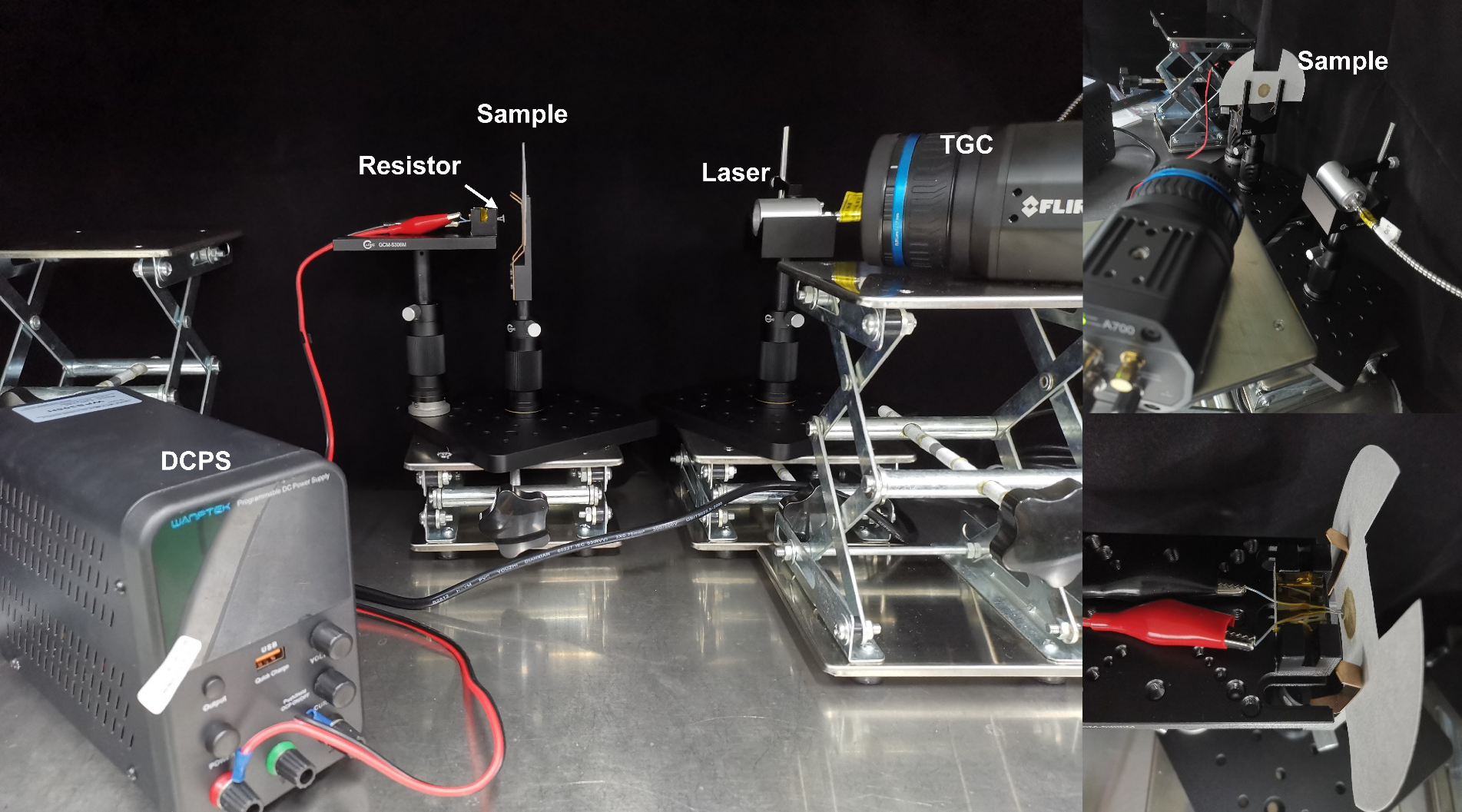


**Fig. S2.** Photographs of setups for measuring LHCE of solid materials.


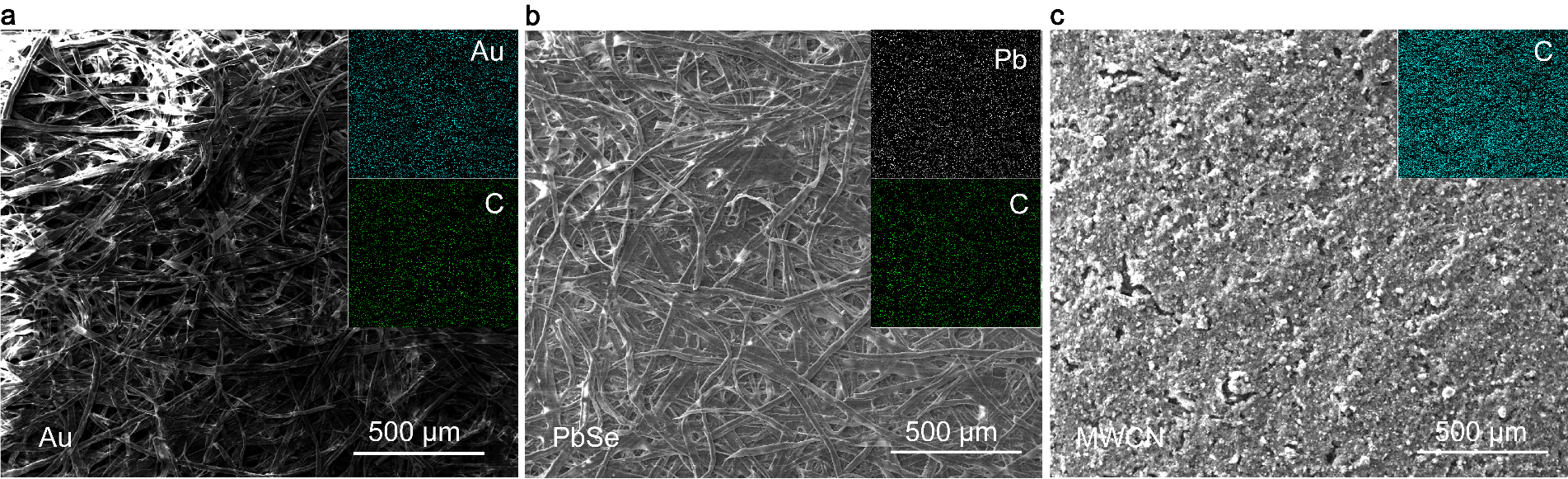


**Fig. S3.** SEM images of Au nanorods (**a**), PbSe nanocrystals (**b**), and MWCN (**c**) on filter paper substrates. The insets are the EDS mapping.


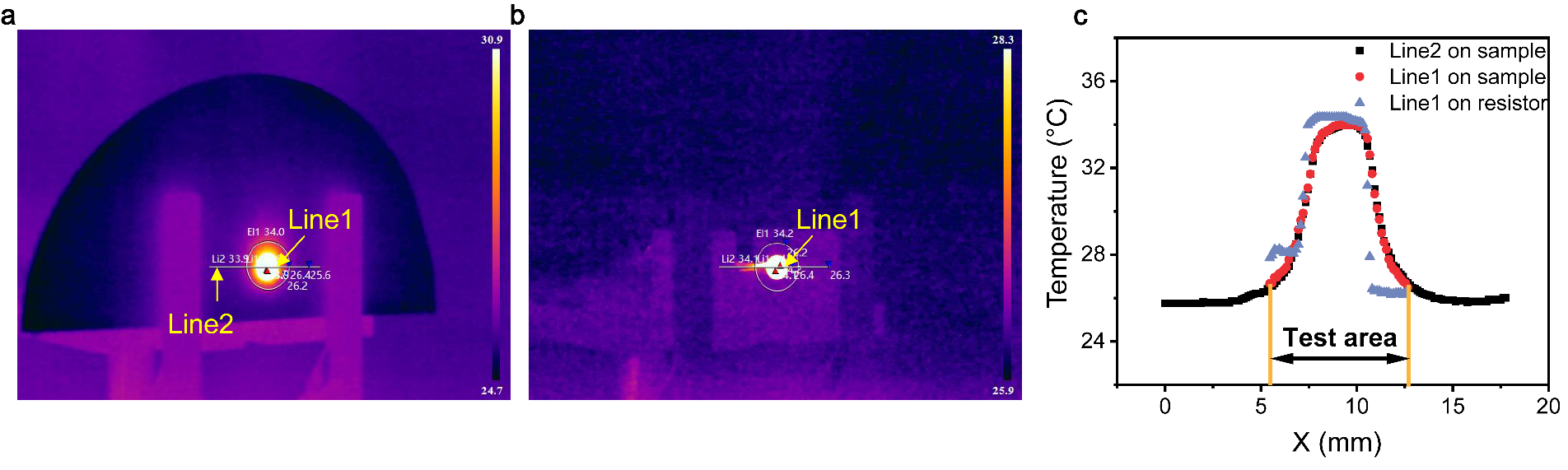


**Fig. S4.** Temperature photos of a sample (**a**) and resistor (**b**) and line distributions of the temperature of Line1 and Line2 (**c**), where Line1 is the diameter of the test area while Line2 is the extension of Line1.


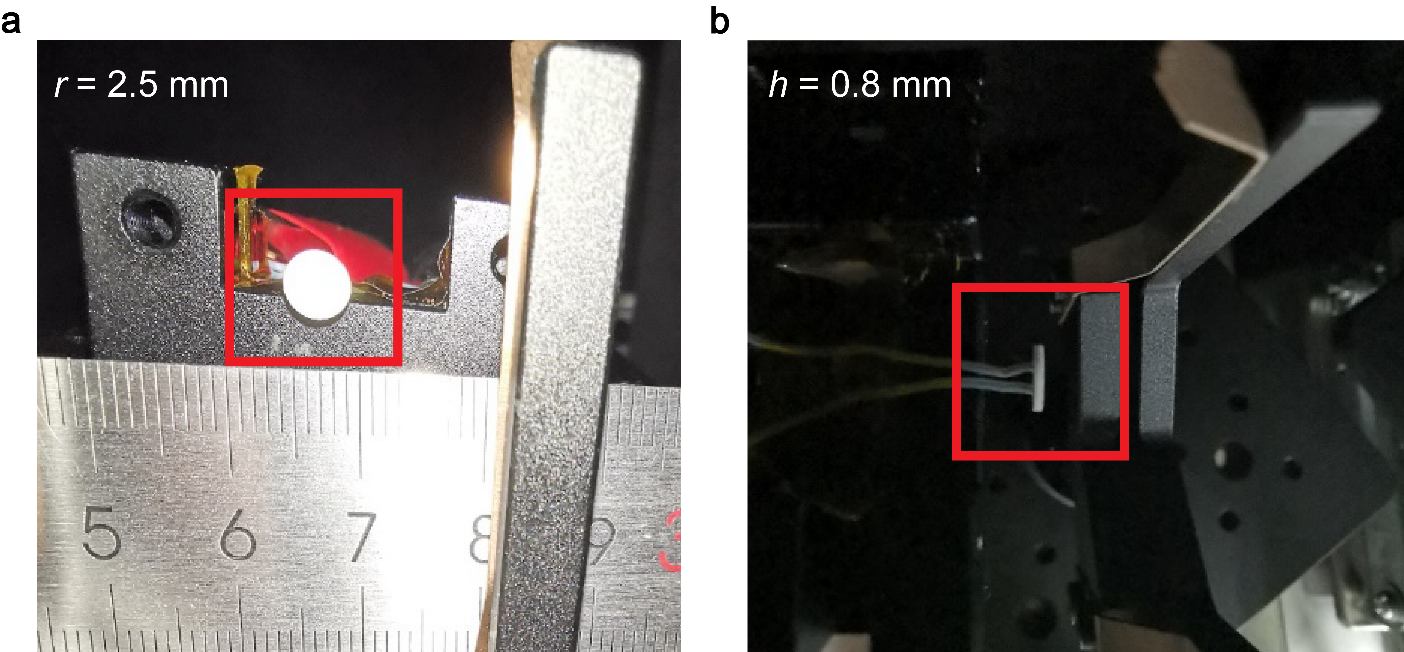


**Fig. S5.** Photographs of the size and shape of the resistor.


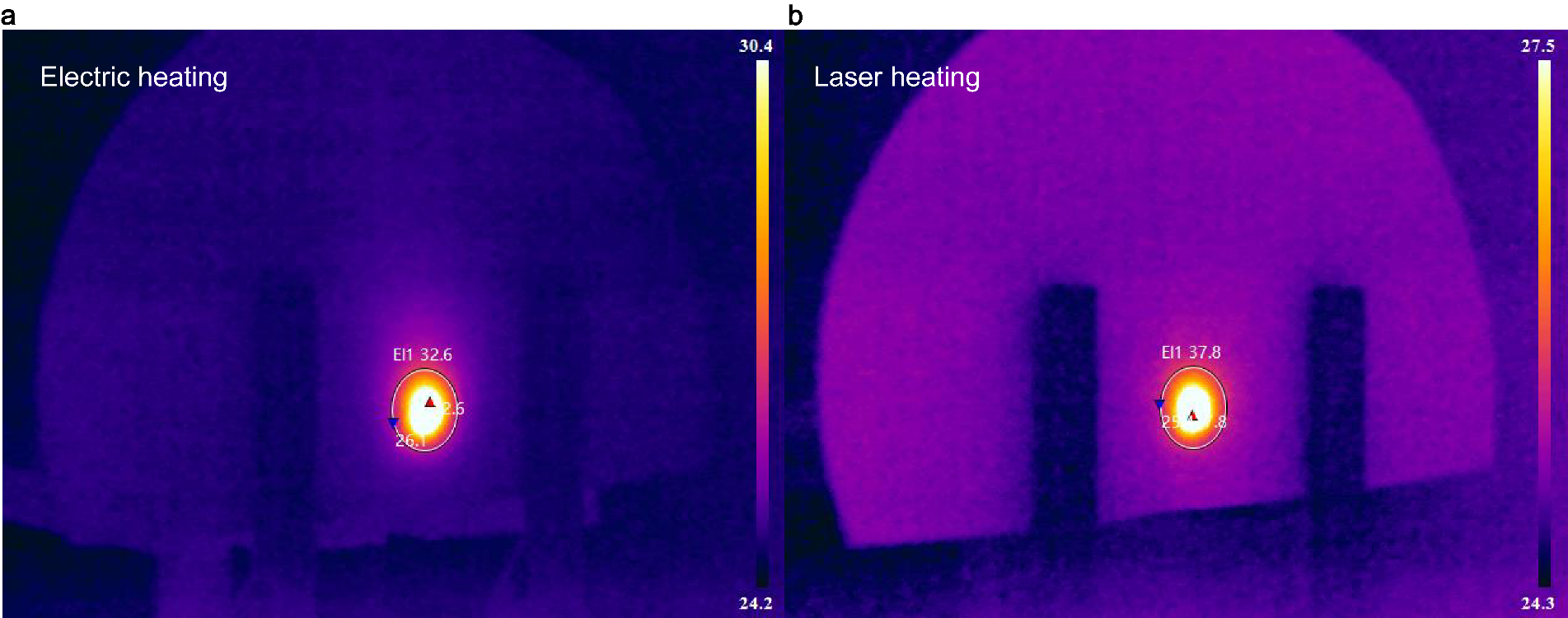


**Fig. S6.** Temperature photographs of a sample under electric (**a**) and laser heating (**b**), where the size and position of the test area are fixed.


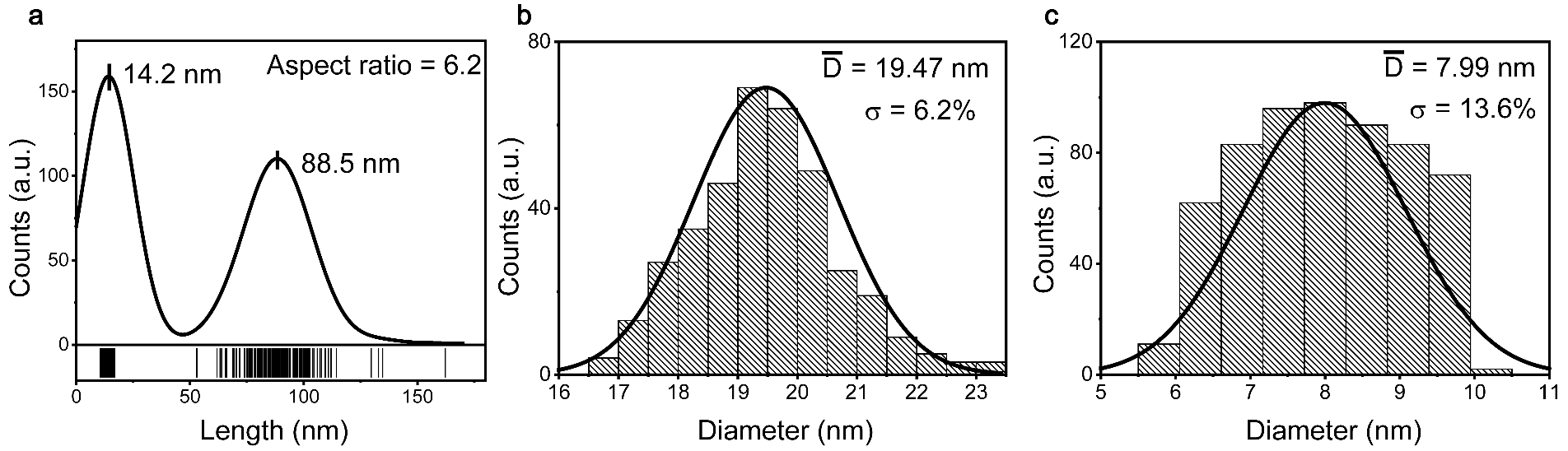


**Fig. S7.** Size distributions of Au nanorods (**a**), PbSe nanocrystals (**b**) and Cu_2_Se nanocrystals (**c**).


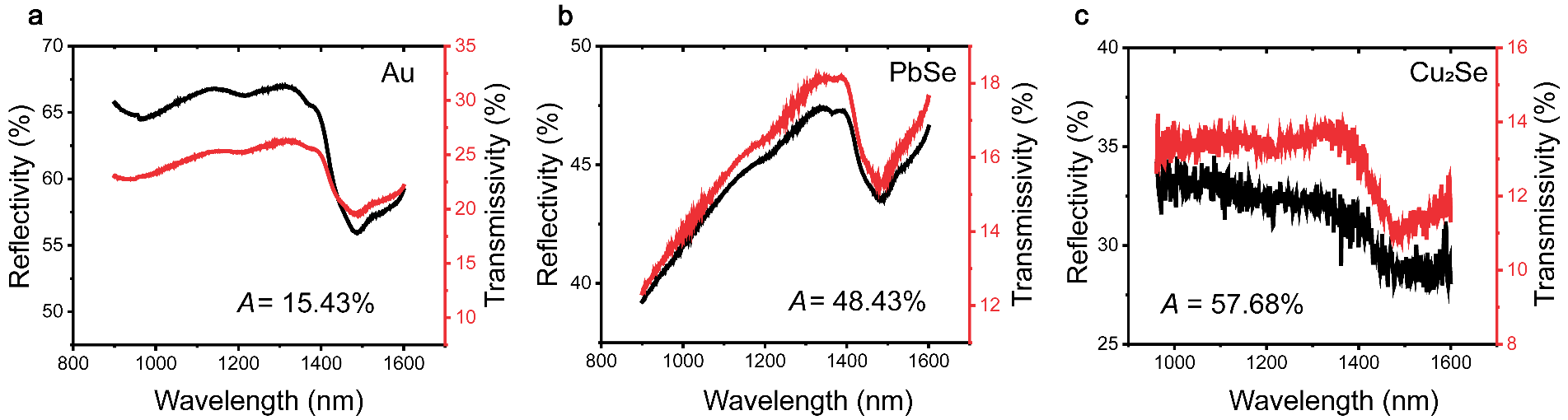


**Fig. S8.** Transmission and reflection spectra of commercial Au nanorods (**a**), PbSe nanocrystals (**b**) and Cu_2_Se nanocrystals (**c**) together with a filter paper.


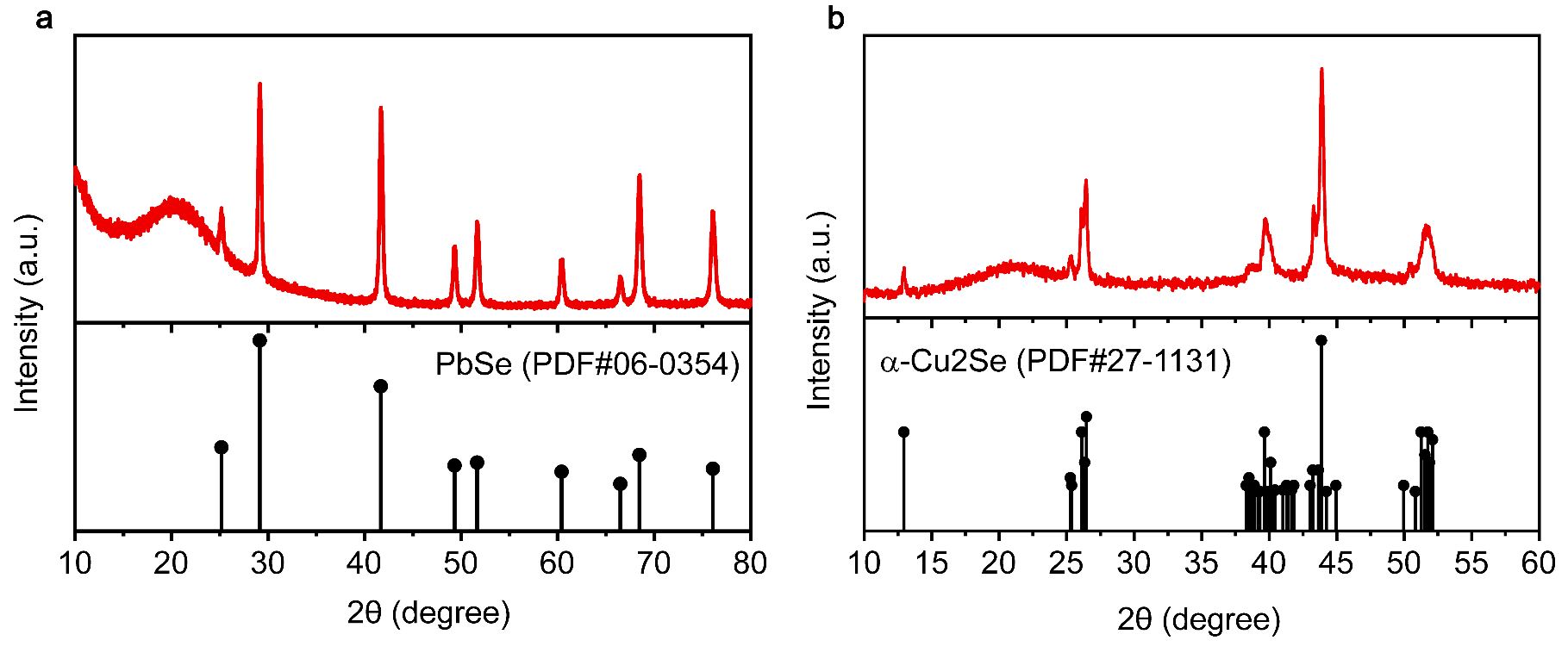


**Fig. S9.** XRD patterns of the synthesized PbSe nanocrystals (**a**) and Cu_2_Se nanocrystals (**b**).


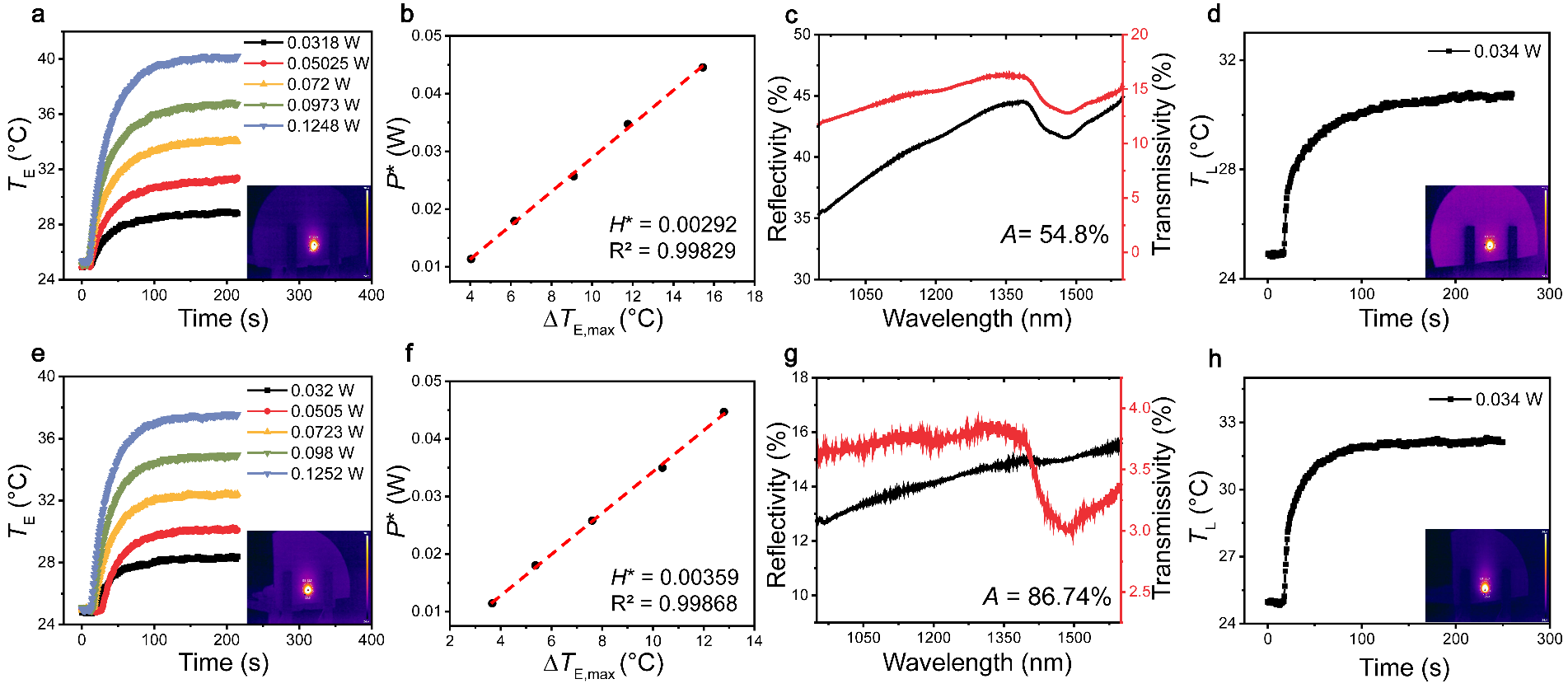


**Fig. S10.** Calculation processes of the LHCE of GO (**a-d**) and graphene (**e-h**).


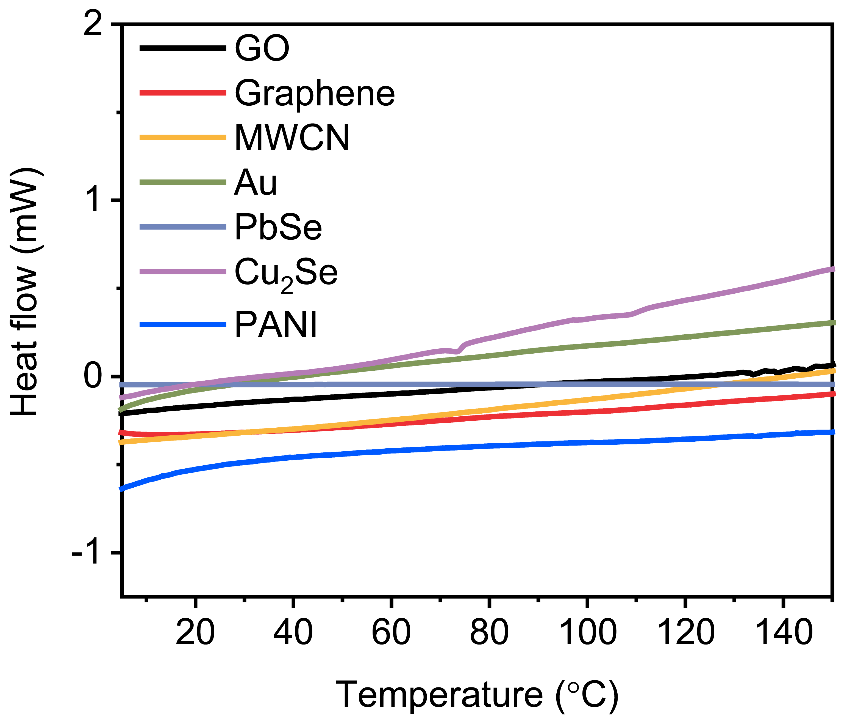


**Fig. S11.** DSC curves for all the samples, where no phase change occurred between the test temperatures of 25 and 60 ℃.


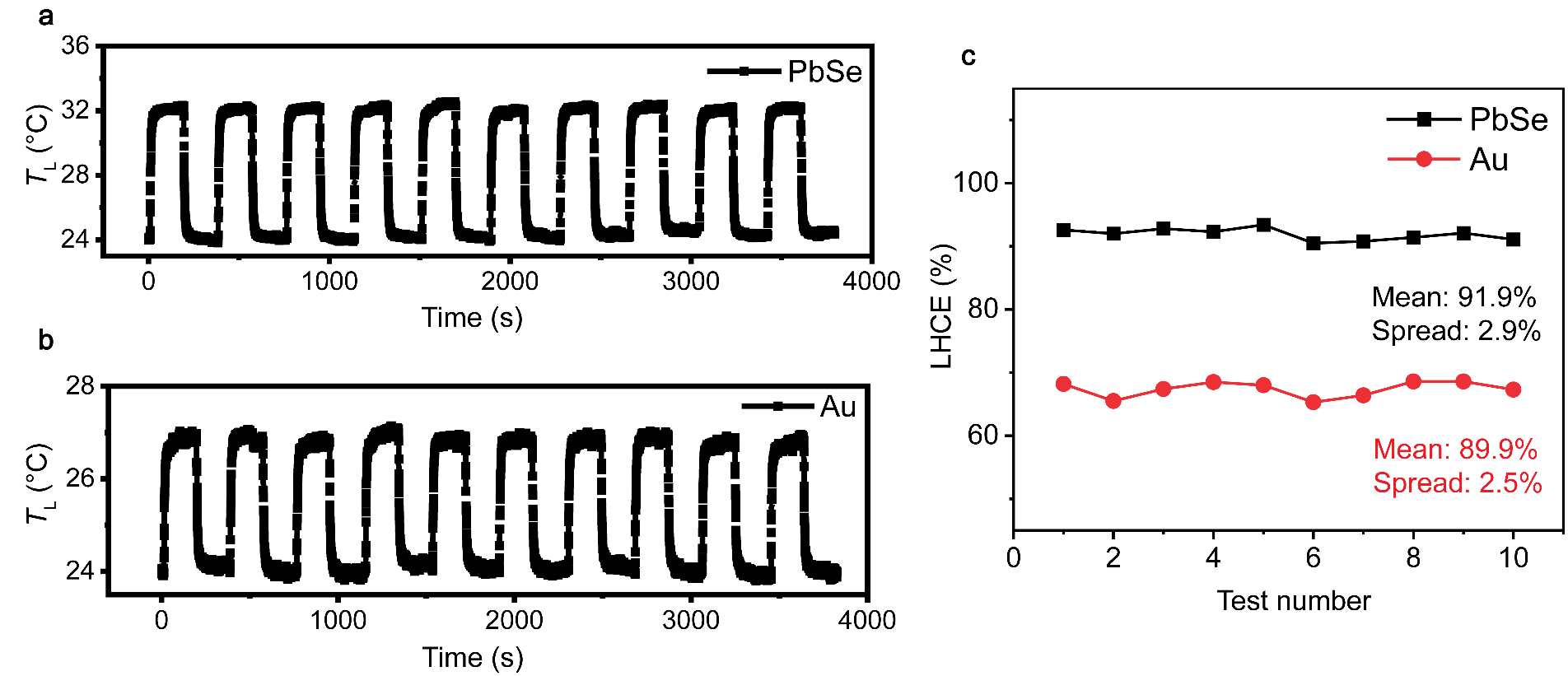


**Fig. S12**. Temperature evolution of PbSe (**a**) and Au (**b**) under continuous laser heating and the corresponding calculated LHCE (**c**)


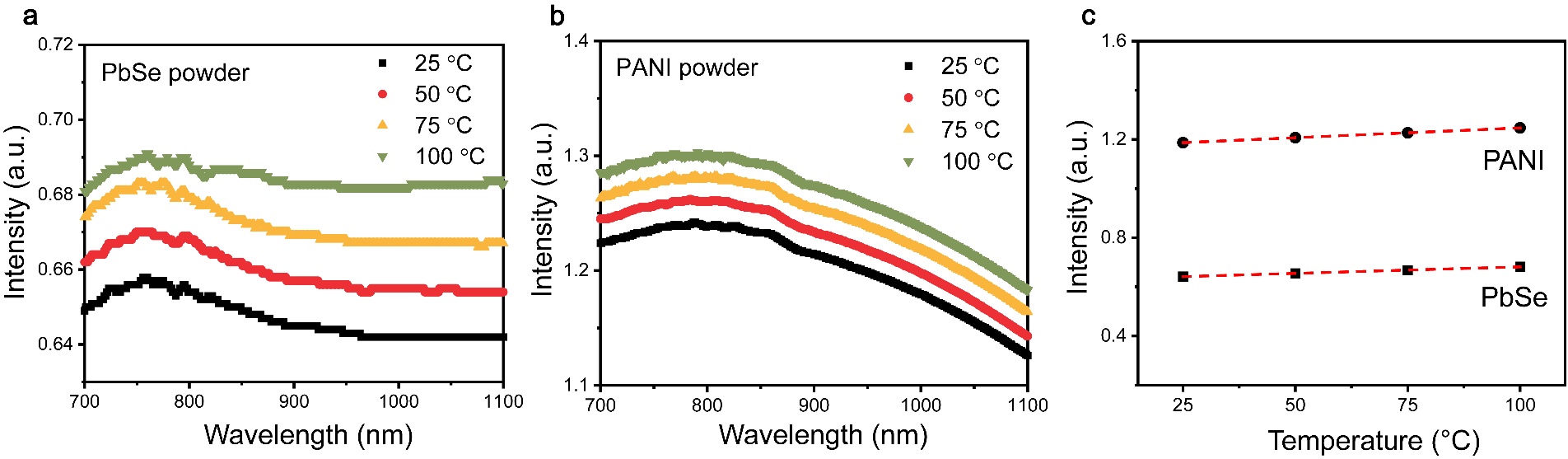


**Fig. S13**. Temperature-dependent absorbance spectra of PbSe powder (**a**) and PANI powder (**b**). (**c**) Absorbance value of PbSe and PANI powder at 980 nm as a function of temperature.


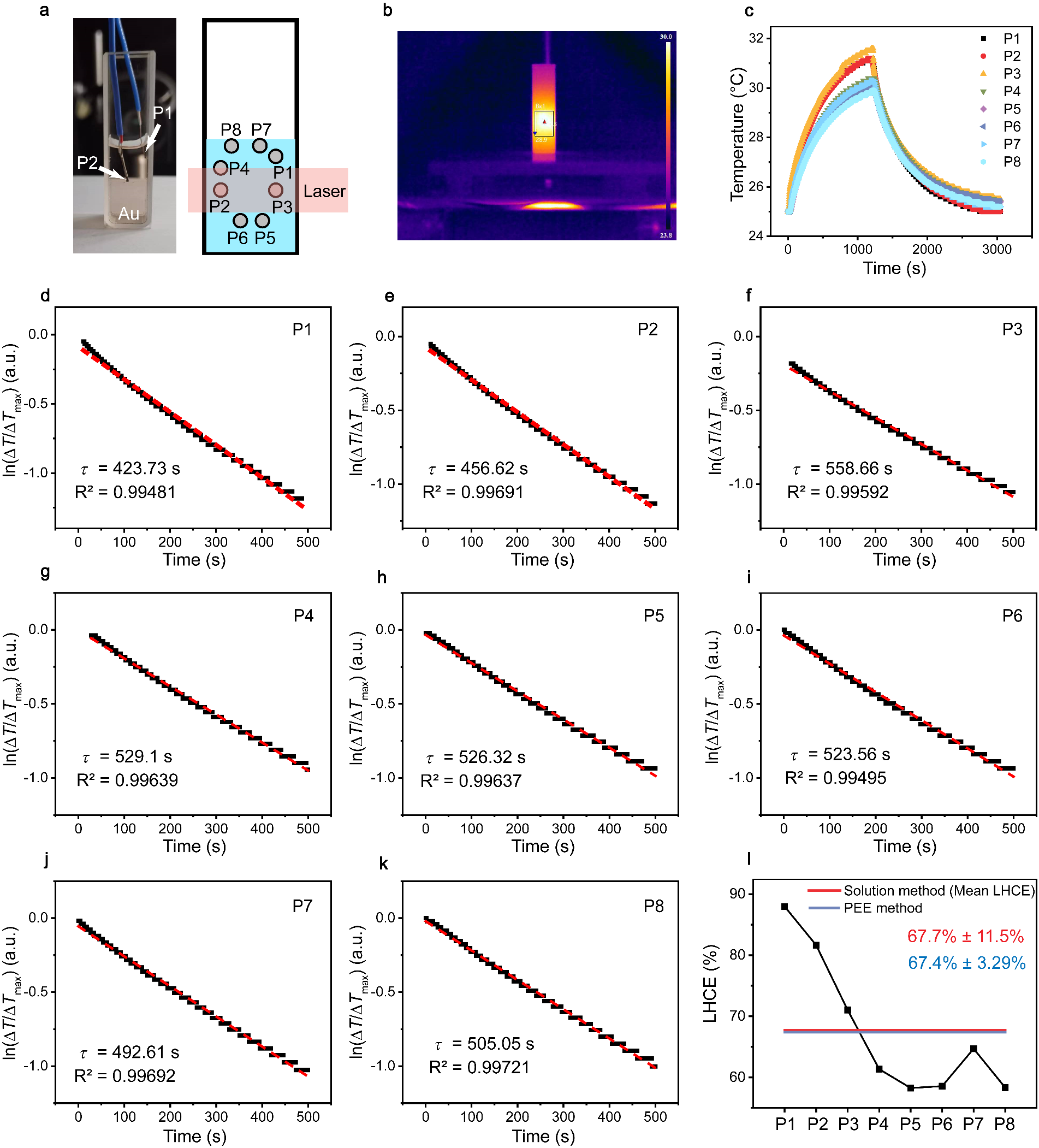


**Fig. S14.** (**a**) Photograph (left panel) and schematic diagram (right panel) of the setups for the comparison experiment, where P1, P2 and P3 etc. represent the positions of the thermocouples. (**b**) Temperature photo of gold nanorods aqueous solution under laser heating. Temperature evolution curves (**c**) and the corresponding cooling curves (**d-k**) of the different thermocouples, where *τ* is the inverse of the slope of the fitted line. (**l**) Calculated LHCE of gold nanorods using solution method and PEE method.


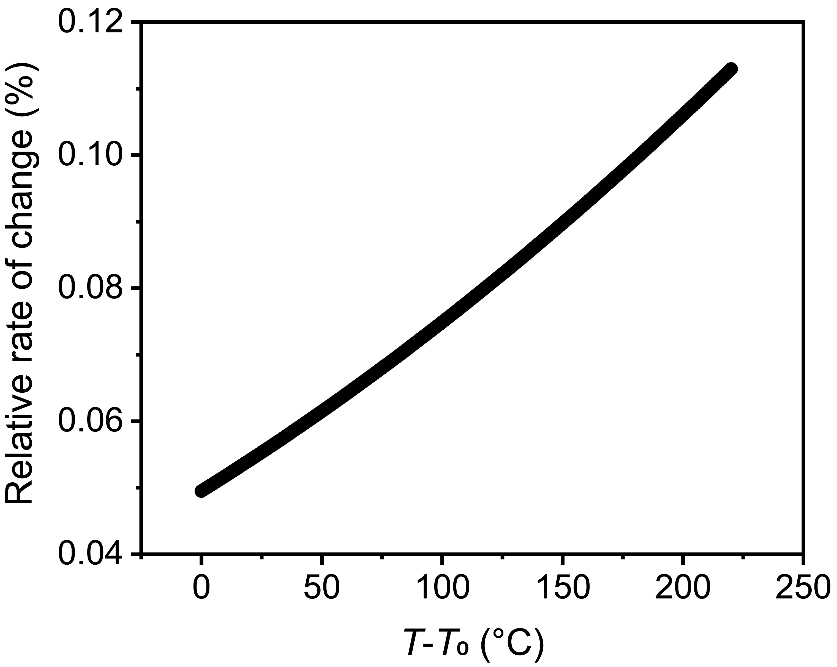


**Fig. S15.** The relative rate of change for *H** as a function of temperature difference.


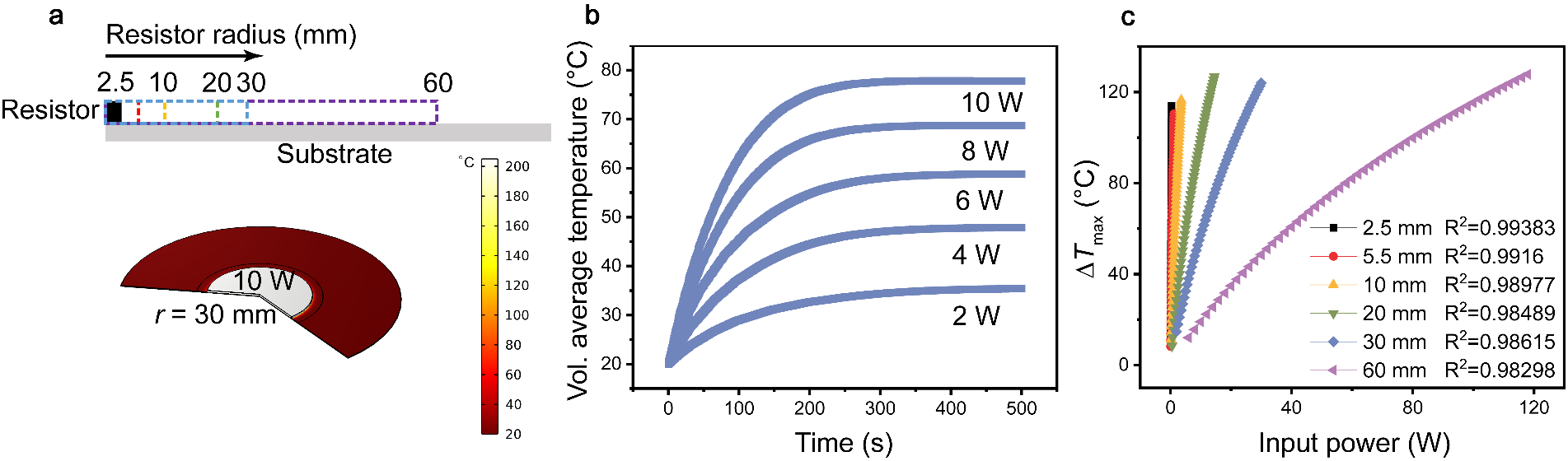


**Fig. S16.** (**a**) Cross-sectional view of and 3D temperature distribution of the simulation model, where the radius of the resistor is variable (2.5 mm to 60 mm), and the radius of the test area is 1.2 times larger than that of the resistor, and the radius of the substrate is a constant value. (**b**) Volume average temperature variation curves of the test area at different input powers with a resistor radius of 30 mm. (**c**) The maximum temperature variations of the test area with different resistor sizes under different input powers. Each curve consists of 30 data points, and the maximum temperature change is almost the same.


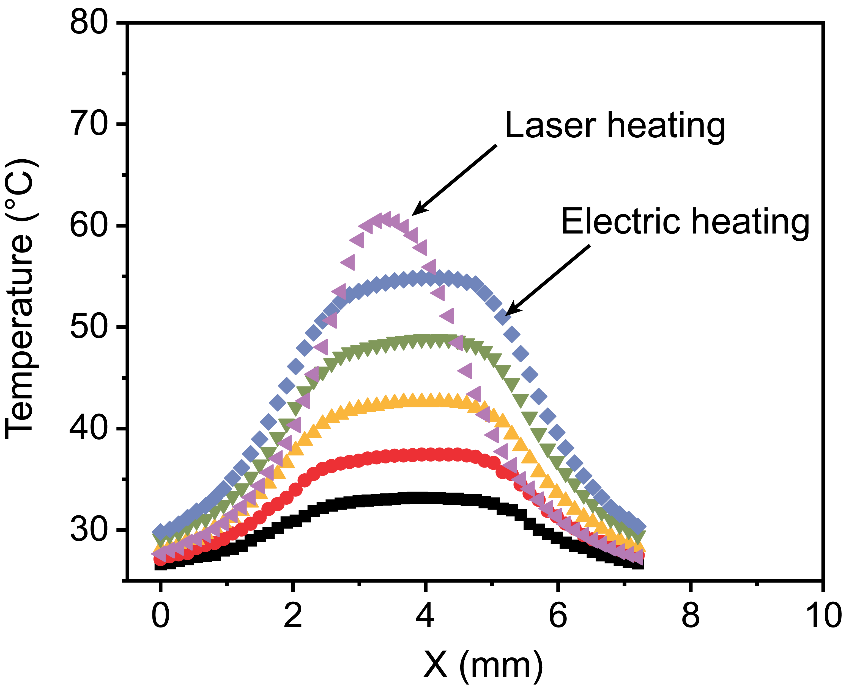


**Fig. S17.** The line temperature distribution of the MWCN sample along the X-direction with different electric and laser powers.


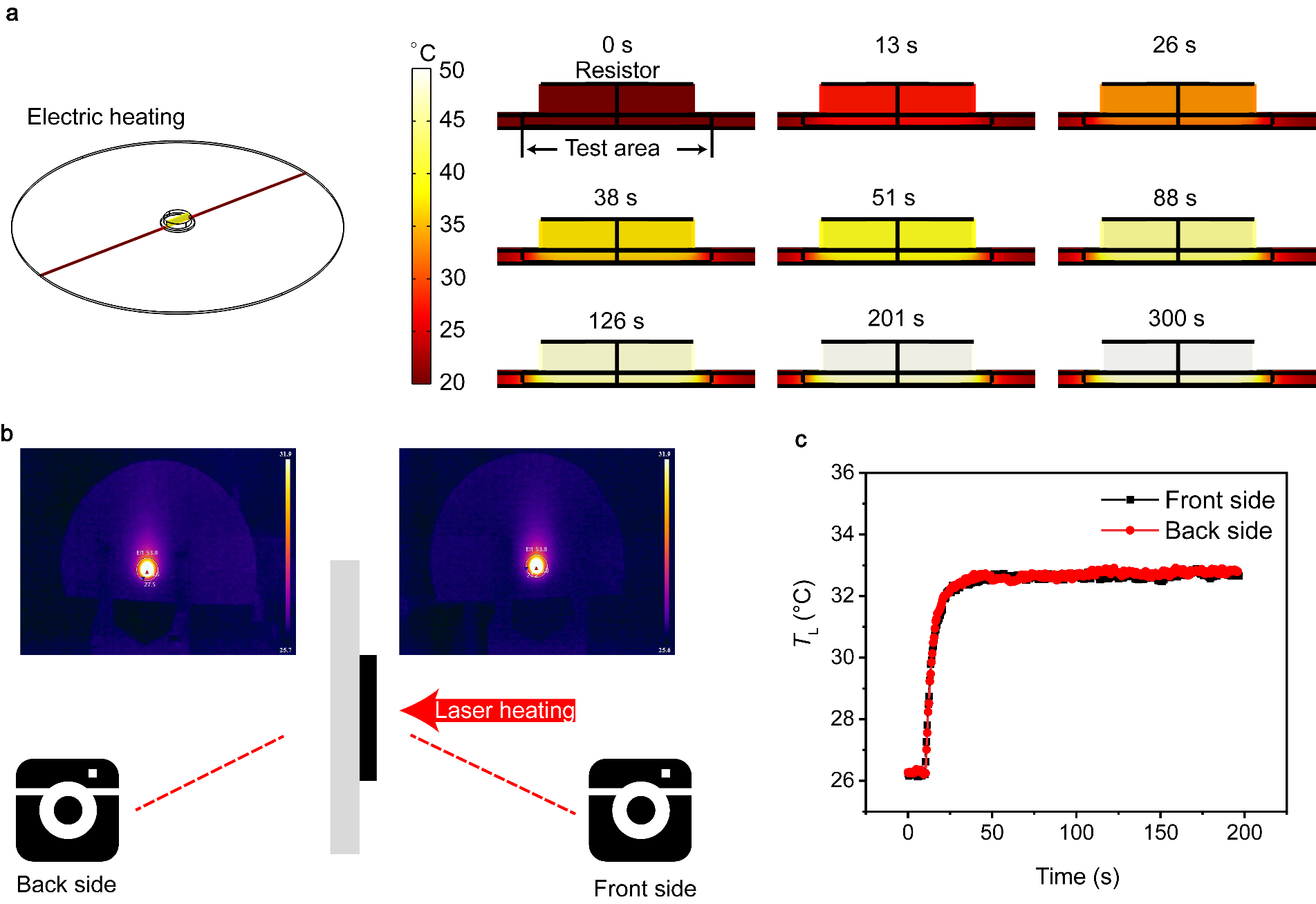


**Fig. S18.** (**a**) Simulation of the temperature distribution of the cross section at different times under electric heating. (**b**) Schematic diagram and temperature photos of TGC measuring the temperature of PbSe sample on the front and back side under laser heating. (**c**) Temperature evolution curves of the front and back side.


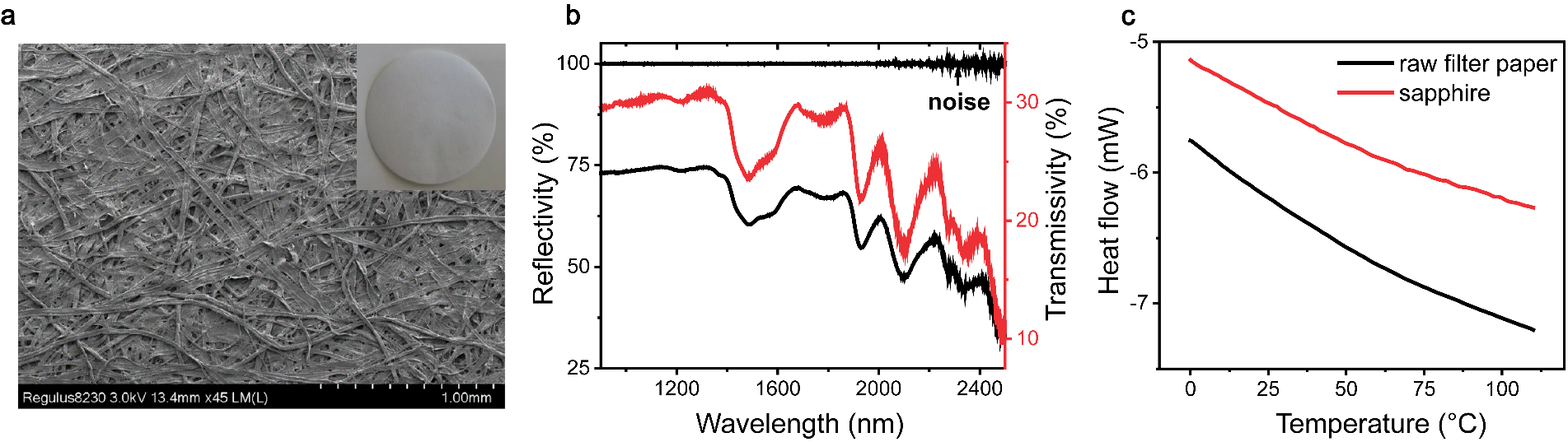


**Fig. S19.** (**a**) SEM image of a raw filter paper and the inset is a photograph of the raw filter paper. (**b**) Transmission and reflection spectra of the raw filter paper. (**c**) DSC curves of the raw filter paper and the standard sapphire reference.


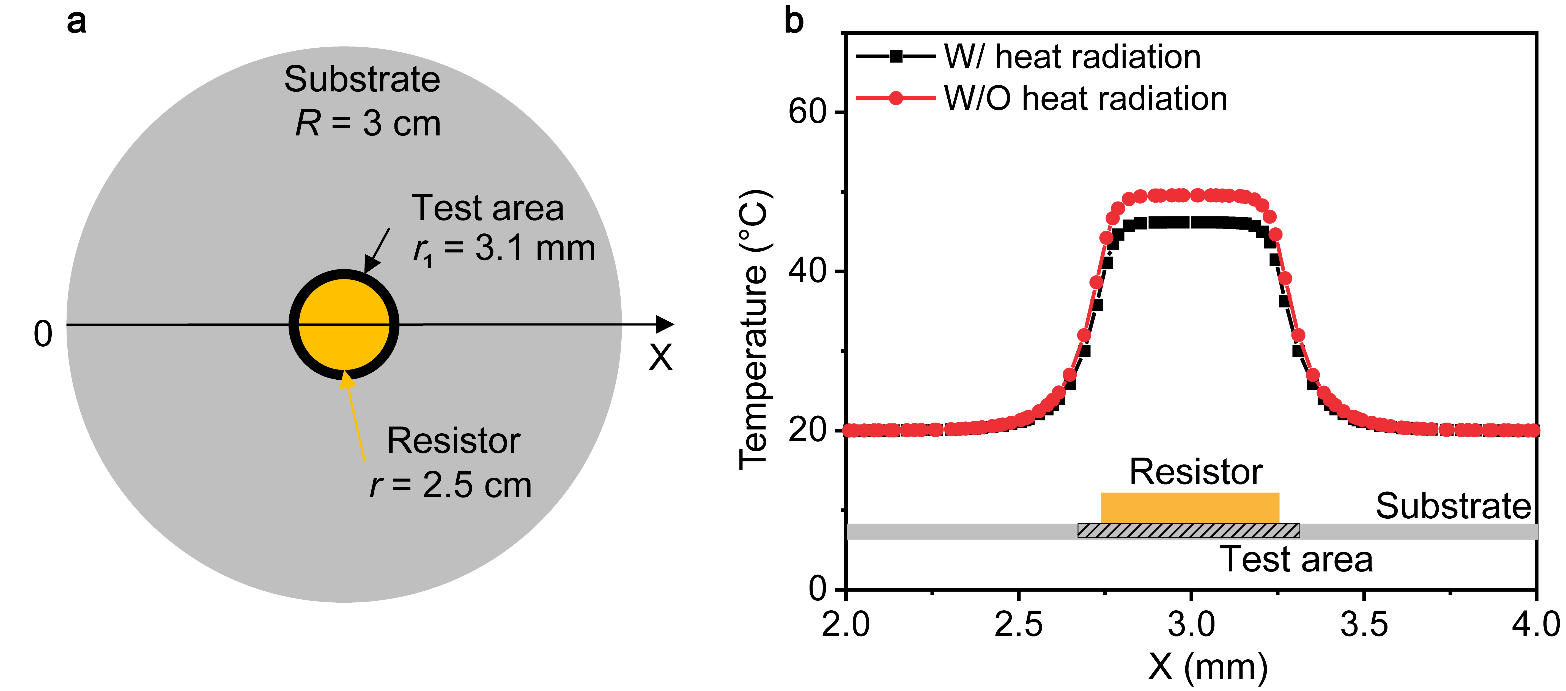


**Fig. S20.** (**a**) Schematic diagram of simulation modeling. (**b**) Simulated temperature line distribution along the X-direction.

**References**

1 Roper, D. K., Ahn, W. & Hoepfner, M. Microscale heat transfer transduced by surface plasmon resonant gold nanoparticles. The Journal of Physical Chemistry C 111, 3636-3641 (2007).

2 Richardson, H. H. *et al*. Experimental and theoretical studies of light-to-heat conversion and collective heating effects in metal nanoparticle solutions. *Nano Letters* **9**, 1139-1146 (2009).

3 Wang, X. C. *et al*. Understanding the photothermal effect of gold nanostars and nanorods for biomedical applications. *RSC Advances* **4**, 30375-30383 (2014).

4 Paściak, A. *et al*. Standardization of methodology of light-to-heat conversion efficiency determination for colloidal nanoheaters. *ACS Applied Materials & Interfaces* **13**, 44556-44567 (2021).

5 Baffou, G. *et al*. Photoinduced Heating of Nanoparticle Arrays. *ACS Nano* **7**, 6478–6488 (2013).

6 Amendola, V. *et al*. Surface plasmon resonance in gold nanoparticles: a review. *Journal of Physics: Condensed Matter* **29**, 203002 (2017).

7 Yeshchenko O.A. *et al*. Temperature dependence of the surface plasmon resonance in gold nanoparticles. *Surface Science* **608**, 275-281 (2013).

8 Bouillard J. G. *et al*. Low-Temperature Plasmonics of Metallic Nanostructures. *Nano Letters* **12**, 1561-1565 (2012).

9 Wu S. S. *et al*. Temperature-Dependent Optical Properties of Graphene on Si and SiO_2_/Si Substrates. *Crystals* **11**, 358 (2021).
